# Niacin Derivatives in MASLD: Metabolic and Therapeutic Insights

**DOI:** 10.3390/nu18060996

**Published:** 2026-03-20

**Authors:** Marina Idalia Rojo-López, Julia Niño-Narvión, Maria Antentas, Berta Fernández-Camins, Elizabeth Martínez-Rojo, Maria Poca, María Antonia Martínez-Sánchez, Bruno Ramos-Molina, Joana Rossell, Didac Mauricio, Josep Julve

**Affiliations:** 1 Research Group in Endocrinology, Diabetes and Nutrition, Institut de Recerca Sant Pau, 08041 Barcelona, Spain; nut.marina.rojo.l@gmail.com (M.I.R.-L.); jnino@santpau.cat (J.N.-N.); maantentas@gmail.com (M.A.); fernandezcamins@gmail.com (B.F.-C.); jrossell@santpau.cat (J.R.); 2 DAP-Cat Group, Unitat de Suport a la Recerca Barcelona, Fundació Institut Universitari per a la Recerca a l’Atenció Primària de Salut Jordi Gol i Gurina (IDIAPJGol), 08007 Barcelona, Spain; 3 Escola de Doctorat, University of Vic—Central University of Catalonia, 08500 Vic, Spain; 4 Genetic Research Unit, Faculty of Chemistry, Autonomous University of Querétaro, Querétaro 76010, Mexico; elizaro4309@gmail.com; 5 Department of Digestive Pathology, Hospital de la Santa Creu i Sant Pau, 08041 Barcelona, Spain; mpoca@santpau.cat; 6 Spanish Biomedical Research Center in Hepatic Diseases (CIBEREHD), Instituto de Salud Carlos III, 28029 Madrid, Spain; 7 Obesity, Diabetes and Metabolism Research Group, Biomedical Research Institute of Murcia (IMIB), 30120 Murcia, Spain; mariaantonia.martinez1@gmail.com (M.A.M.-S.); bruno.ramos@imib.es (B.R.-M.); 8 Spanish Biomedical Research Center in Diabetes and Associated Metabolic Diseases (CIBERDEM), Instituto de Salud Carlos III, 28029 Madrid, Spain; 9 Faculty of Medicine, University of Vic—Central University of Catalonia, 08500 Vic, Spain; 10 Department of Endocrinology and Nutrition, Hospital de la Santa Creu i Sant Pau, 08041 Barcelona, Spain

**Keywords:** hepatic steatosis, MASLD, nicotinamide, vitamin B3, MASH, hepatic fibrosis, inflammation, regeneration, sirtuin, oxidative stress, mitochondria, NAD^+^ precursors, alcohol-induced liver disease

## Abstract

Metabolic dysfunction-associated steatotic liver disease (MASLD) is becoming increasingly prevalent worldwide, particularly among individuals with obesity and type 2 diabetes (T2D). MASLD remains potentially reversible in the early phases but, without timely intervention, it can progress to metabolic dysfunction-associated steatohepatitis (MASH) and hepatic fibrosis, which in turn may advance to cirrhosis and hepatocellular carcinoma over time. With no pharmacological treatments specifically indicated for MASLD, current therapeutic strategies include lifestyle modifications, including dietary modifications. Niacin and its molecular derivatives (collectively belonging to the vitamin B3 group) play a central role in metabolic processes, especially through their involvement in the biosynthesis of the oxidized form of nicotinamide adenine dinucleotide (NAD^+^). A growing body of preclinical evidence suggests that reduced NAD^+^ levels are a hallmark of MASLD, and that NAD^+^ precursors may help attenuate disease progression through multiple mechanisms, including sirtuin 1 (SIRT1)-mediated inhibition of hepatic lipogenesis. Although these findings from experimental models suggest a potential role for niacin and related molecular derivatives as a modulators of MASLD-related pathways, evidence from human studies remains limited and inconsistent. For instance, interventional studies evaluating niacin or molecular derivatives supplementation have reported variable findings, with several trials showing limited meaningful benefits on MASLD-related outcomes. Consequently, further well-designed, controlled trials are needed to clarify therapeutic efficacy, dose–response relationship, and the feasibility of integrating niacin derivatives into dietary or therapeutic strategies aimed at reducing liver fat and improving adverse metabolic outcomes. This review aims to (i) summarize mechanistic insights on the role of niacin as a source of NAD^+^ on experimental MASLD and (ii) critically evaluate the available human evidence on the effect of supplemental niacin and derivatives in the prevention of MASLD development and its progression to MASH and fibrosis.

## 1. Introduction

Metabolic dysfunction-associated steatotic liver disease (MASLD) represents the leading cause of chronic liver disease worldwide, affecting up to one third of adults globally. The growing burden is closely linked to the rising prevalence of obesity, metabolic syndrome, and type 2 diabetes mellitus (T2D) [1]. MASLD is characterized by excessive hepatic triglyceride storage in the liver without significant alcohol intake and with at least one cardiometabolic risk factor [1]. The disease encompasses a spectrum from simple hepatic steatosis to more advanced stages, including metabolic dysfunction-associated steatohepatitis (MASH), fibrosis, cirrhosis, and hepatocellular carcinoma. Beyond liver-specific complications, MASLD contributes substantially to cardiovascular and metabolic morbidity [2,3,4]. Current management strategies for MASLD primarily rely on lifestyle interventions, particularly dietary modification and increased physical activity [5,6,7,8,9]. There are no pharmacotherapies specifically indicated for MASLD, and once MASLD progresses to end-stage liver disease, liver transplantation may be the only option. Resmetirom, a THR-β agonist, has recently been approved for MASH and the glucagon-like peptide-1 (GLP-1) receptor agonist semaglutide, indicated for T2D and for obesity, has also shown promise for MASH [10,11]. Nevertheless, there is still an unmet need for effective and tolerable interventions to help address the mounting challenge in preventing the onset and progression of MASLD.

Nicotinamide adenine dinucleotide (NAD^+^) is a key regulator of cellular energy metabolism. Declining NAD^+^ levels have been associated with aging and numerous pathological conditions, especially those involving mitochondrial and metabolic dysfunction [12]. As such, there has been interest in the restoration of NAD^+^ levels as a therapeutic option for a variety of human diseases [13]. However, the strength of evidence varies across mechanisms, particularly in human experimental models and human studies.

Niacin (also known as vitamin B3, nicotinic acid) along with nicotinamide (NAM) and its derivatives are precursors of NAD^+^ [14]. Because these molecules are available naturally in many foods, and in vitamin and dietary supplements [14], it has been suggested that their deficiency may not generally stem from nutritional deficits, but rather from metabolically adverse conditions that contribute to the depletion of hepatic NAD^+^ content in MASLD [15]. This has highlighted the need for intervention studies using pharmacological doses to counteract pathological states [16]. However, the direct therapeutic contribution of niacin and NAM derivatives in human MASLD remains inconclusive, likely due, at least in part, to differences in dosage, treatment duration, or disease stage across studies. Therefore, several relevant gaps remain in the context of MASLD. First, previous reviews have not systematically integrated observational evidence on dietary niacin intake with interventional studies using pharmacological doses or specific NAD^+^ precursors, leaving it unclear whether habitual intake and supplementation exert comparable or divergent effects. Second, the literature has rarely examined differences across vitamin B3 forms (niacin, NAM, and related derivatives) within a unified translational framework. Third, several recently published studies, including novel analyses of dietary niacin intake in relation to MASLD risk, have not yet been collectively synthesized. This review aims to address these gaps by providing an updated and critical comparison of observational and interventional evidence, thereby clarifying the translational relevance of niacin and related NAD^+^ precursors in MASLD. However, the extent to which these mechanistic observations from experimental models translate to human MASLD remains incompletely understood.

## 2. Methodology

### 2.1. Search Strategy

The search strategy for this review was carried out between August 2025 and September 2025 using the PubMed, Web of Science, and Scopus databases. The search terms included were combined using Boolean operators as follows: “nicotinamide” OR “nicotinamide riboside” OR “niacin” OR “vitamin B3” AND “liver fibrosis” OR “liver steatosis” OR “nonalcoholic fatty liver disease” OR “NAFLD” OR “non-alcoholic steatohepatitis” OR “NASH” OR “metabolic dysfunction-associated steatotic liver disease” OR “MASLD” OR “metabolic dysfunction-associated steatohepatitis” OR “MASH”.

### 2.2. Inclusion Criteria

Studies were included if they were original in vitro, in vivo, observational, or clinical intervention studies evaluating the effects or intake of niacin, NAM, nicotinamide riboside (NR), or nicotinamide mononucleotide (NMN). Eligible in vitro studies used hepatic or hepatic stellate cell models (human or murine), applied validated stimuli to induce steatosis, inflammation, lipotoxicity, endoplasmic reticulum (ER) stress, or fibrosis, and reported molecular or cellular outcomes. In vivo studies included mammalian models of diet- or toxin-induced steatosis, steatohepatitis, metabolic dysfunction, or fibrosis, with defined dosing regimens and hepatic endpoints. Observational human studies were required to assess dietary niacin using validated recalls or FFQs and to report liver-related outcomes. Clinical trials had to specify population characteristics, dosing, duration, adherence monitoring, and hepatic or metabolic endpoints.

### 2.3. Exclusion Criteria

Exclusion criteria comprised reviews, commentaries, conference abstracts, non-mammalian models, studies not assessing the molecules of interest, lack of validated dietary assessment (in observational work), insufficient methodological detail, unavailable full text, or absence of liver-related outcomes.

## 3. NAD^+^ Metabolism in the Liver

Hepatocytes accumulate most of their NAD^+^ within mitochondria, where most cellular oxidation-reduction reactions occur [17,18]. However, NAD^+^ is also present in the cytosol and the nucleus, where NAD^+^-derived metabolites and NAD^+^-dependent enzymes contribute to numerous cellular functions, including transcriptional regulation, DNA repair, and signaling pathways [14,17,18]. Recent years have seen significant progress in understanding tissue-specific NAD^+^ synthesis, transport, and catabolism.

**Figure 1:**
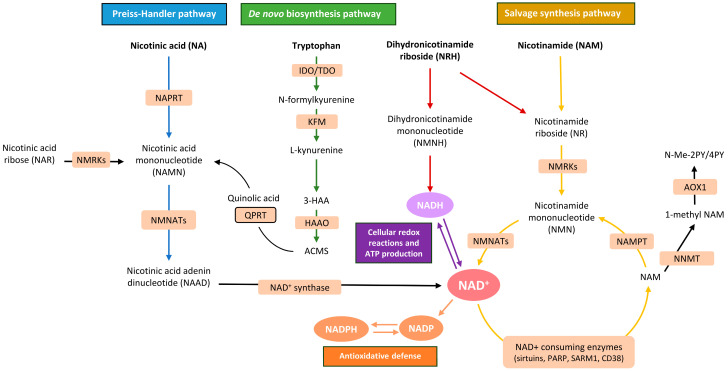
NAD^+^ metabolism in the liver. Mammalian cells synthesize NAD^+^ de novo from tryptophan via the kynurenine pathway, or from nicotinic acid (niacin) through the Preiss–Handler pathway. Most NAD^+^ can be regenerated via salvage pathways from NAM and NR, which form NMN, a key intermediate. NAD^+^ can also be synthesized from dihydronicotinamide riboside (NRH), a reduced form of NR. NAD^+^ supports redox reactions by cycling with NADH in glycolysis, fatty acid β-oxidation, and the tricarboxylic acid cycle, and contributes to ATP production via oxidative phosphorylation, by using energy released from electrons transferred through the electron transport chain in mitochondria. Additionally, NAD^+^ serves as a co-substrate for enzymes such as PARPs, sirtuins, CD38, and SARM1, thereby influencing different cellular processes, i.e., DNA repair, metabolism, gene regulation, and inflammation [19,20,21]. This figure was adapted from information reported in references [17,18]. Abbreviations: ACMS, α-amino-β-carboxymuconate-ε-semialdehyde; AOX1, aldehyde oxidase 1; CD38, cluster of differentiation 38; HAAO, 3-hydroxyanthranilate; 3-HAA, 3,4-dioxygenase; IDO/TDO, indoleamine 2,3-dioxygenase/tryptophan 2,3-dioxygenase; KFM, kynurenine formamidase; NA, nicotinic acid; NAAD, nicotinic acid adenine dinucleotide; NAD^+^, nicotinamide adenine dinucleotide (oxidized form); NADH, nicotinamide adenine dinucleotide (reduced form); NAM, nicotinamide; NAMN, nicotinic acid mononucleotide; NAMPT, nicotinamide phosphoribosyltransferase; NAPRT, nicotinate phosphoribosyltransferase; NAR, nicotinic acid ribose; *N*-Me-2PY/4PY, *N*-methyl-2-pyridone-5-carboxamide, *N*-methyl-4-pyridone-3-carboxamide; NMN, nicotinamide mononucleotide; NMNATs, nicotinamide mononucleotide adenylyltransferases; NMRKs, nicotinamide riboside kinase; NNMT, nicotinamide *N*-methyltransferase; NR, nicotinamide riboside; PARP, Poly (ADP-ribose) polymerases; QPRT, quinolinate phosphoribosyltransferase; SARM1, sterile alpha and TIR motif containing 1.

In mammalian cells, NAD^+^ can be synthesized through three principal pathways: de novo synthesis from tryptophan, the Preiss–Handler pathway from niacin, and the salvage pathway, which recycles breakdown products such as NAM and NR back into NAD^+^, ensuring its continuous supply (Figure 1). Whereas most tissues predominantly use the salvage pathway, the liver, and to a lesser extent the kidney, can synthesize NAD^+^ from the amino acid tryptophan via the kynurenine (de novo) pathway, which contains all the enzymes necessary for NAD^+^ de novo biosynthesis (Figure 1) [17,18].

### 3.1. NAD^+^ Synthesis

Hepatocytes play a central role in NAD^+^ metabolism, as they can synthesize NAD^+^ from multiple precursors via both the de novo and salvage pathways [22,23]. Nevertheless, hepatocytes primarily generate NAD^+^ from preformed pyridine moieties, such as NAM, which represents the most efficient route for NAD^+^ biosynthesis. NAM can be available intracellularly as an end-product of non-oxidative NAD^+^ catabolism and therefore serves as a readily available substrate for NAD^+^ production via nicotinamide phosphoribosyltransferase (NAMPT), the rate-limiting enzyme of the NAD^+^ salvage pathway (Figure 1) [24,25].

Intracellular recycling of NAD^+^ is, however, limited because NAM is regularly metabolized and excreted in urine. Thus, dietary intake of NAD^+^ precursors, including NAM, niacin, NMN, and NR, collectively known as vitamin B3, is required to sustain organismal NAD^+^ homeostasis [18,26,27]. Key enzymes that enable the salvage pathway of NAD^+^ synthesis from NR and NMN, such as nicotinamide riboside kinases (NRK1/NRK2) and nicotinamide mononucleotide adenylyltransferases (NMNATs), respectively, are essential determinants of how exogenous precursors are converted into NAD^+^ in hepatocytes and other cell types [23,28]. Beyond classical niacin-derived NAD^+^ precursors such as NR and NMN, nicotinamide riboside hydride (NRH), a reduced form of NR, has emerged as a highly potent NAD^+^ precursor, outperforming NR in its capacity to rapidly increase intracellular NAD^+^ levels in preclinical systems. NRH is converted to NAD^+^ through an adenosine kinase-dependent pathway that is distinct from the canonical NAMPT-dependent salvage pathway. However, the functional metabolic and mitochondrial consequences of NRH supplementation, particularly in the context of MASLD, remain to be established [29,30].

### 3.2. NAD^+^ Consumption

NAD^+^ serves as a co-substrate for several NAD^+^-consuming enzymes, including SIRTs, CD38, poly(ADP-ribose) polymerases (PARPs), and sterile alpha and TIR motif-containing 1 (SARM1), thereby regulating key cellular processes in the context of MASLD such as DNA repair, epigenetic and transcriptional control, metabolic homeostasis, and inflammatory signaling [19,20,21].

NAD^+^ is also a mandatory coenzyme for different NAD^+^-consuming enzymes, including SIRTs, a class of NAD^+^-dependent protein deacetylases that are involved in the physiological homeostasis of different organs and systems [31,32,33]. Specifically, SIRT1 governs a wide spectrum of cellular and organelle processes and plays beneficial roles in regulating hepatic lipid metabolism, controlling hepatic oxidative stress and mediating hepatic inflammation at least in part through deacetylating certain transcriptional regulators against the progression of MASLD [34].

A major route of NAD^+^ consumption in liver as in other tissues is via ecto- and intracellular NADases (also mentioned as NAD^+^ hydrolases) such as CD38; CD38 upregulation is implicated in tissue NAD^+^ decline under metabolic stress conditions (including in liver-associated pathologies) and strongly influences both systemic and local NAD^+^ availability [35].

Poly(ADP-ribose) polymerases (PARPs) are nuclear NAD^+^-consuming enzymes that catalyze the transfer of ADP-ribose units to target proteins, a process known as poly-ADP-ribosylation (PARylation) [19,20]. In the liver, PARPs play a central role in DNA repair, genomic stability, and the regulation of stress responses, with emerging evidence implicating diverse PARP family members in metabolic dysfunction-associated steatotic liver disease and other hepatic disorders [19,36]. Overactivation of PARPs in response to extensive oxidative DNA damage can dramatically deplete cellular NAD^+^ pools, disrupt mitochondrial function, and exacerbate hepatocellular injury [37,38]; inhibition of PARPs both restores NAD^+^ levels and improves mitochondrial function and oxidative stress markers in preclinical models of hepatic steatosis and oxidative insult [37].

Sterile alpha and TIR motif-containing 1 (SARM1) is an NAD^+^-consuming enzyme best characterized in neurons as a central mediator of axonal degeneration [39]. Recent preclinical studies indicate that SARM1 is also expressed in hepatocytes, and its activation contributes to NAD^+^ depletion, oxidative stress, and inflammatory responses in the context of MASLD [21]. In murine high-fat-diet models of MASLD, hepatic SARM1 expression is upregulated, and genetic ablation of SARM1 attenuates steatosis, oxidative stress, inflammation, and liver injury, suggesting a contributory role in disease progression [21]. Although direct evidence in human MASLD/MASH is limited, these findings support the concept that SARM1-mediated NAD^+^ consumption may exacerbate hepatic metabolic and inflammatory dysregulation.

### 3.3. Alternative Regulators of Intracellular NAD^+^ Levels

Hepatic methylation of NAM by nicotinamide *N*-methyltransferase (NNMT) represents an important route of NAM utilization. NNMT, which is mainly expressed in the liver [40], catalyzes the transfer of a methyl group from S-adenosylmethionine (SAM) to NAM, generating 1-methylnicotinamide (MNA). MNA can be further metabolized and excreted in urine [41]; increased hepatic NNMT activity diverts NAM away from the salvage pathway, thereby influencing hepatic NAD^+^ pools and one-carbon metabolism [42]. Interestingly, MNA has recently been reported to stabilize *Sirt1* mRNA, thereby enhancing SIRT1 signaling and providing protection against experimental MASLD [42]. Notably, similar NNMT-mediated SIRT1 stabilization has also been observed in other biological contexts, such as prostate cancer, indicating that this regulatory mechanism is not limited to liver disease [43]. However, the precise mechanisms by which NNMT coordinates these effects remain to be fully elucidated. Consistent with the potential protective actions of MNA, an independent study demonstrated that the administration of hydralazine, an effective inhibitor of aldehyde oxidase, to rats with experimentally induced MASLD ameliorated hepatic steatosis [44]. Since aldehyde oxidase is a primary enzyme responsible for hepatic MNA catabolism [45], its inhibition decreases the rapid metabolic clearance of MNA, thereby increasing the hepatic bioavailability of MNA, with consequent improvement of hepatic steatosis [46].

Recent metabolomic tracing studies have shown that a large fraction of orally administered NR and NMN is metabolized in the gut and liver (including microbiota-mediated deamidation to niacin), resulting in the formation of enterohepatic pools of niacin that the liver preferentially uses for NAD^+^ synthesis in vivo. The finding that the microbiota plays an important role in metabolizing NAD^+^ precursors highlights the complexity of interpreting the outcomes of NAD^+^ precursor supplementation studies, with inter-individual variation in gut microbiota composition potentially influencing precursor bioavailability and metabolism [47].

## 4. Experimental Models of MASLD: Effects of NAD^+^-Increasing Strategies

Hepatic NAD^+^ content levels are critical for maintaining liver metabolic homeostasis [48,49,50], and their reduction has been linked to disrupted hepatic physiology [51,52,53,54,55]. Hepatic NAD^+^ decline has been frequently linked to MASLD/MASH [50,55], and hepatic fibrosis [27,56,57,58,59] in experimental models, whereas the restoration of hepatic NAD^+^ content has been proposed as a potential strategy to counteract MASLD progression [18,27,50,53,60,61,62].

Supplementation with NAD^+^ precursors such as niacin, NAM, NR, and NMN has shown promise in exerting hepatoprotective effects in experimental models of MASLD, MASH, and hepatic fibrosis [25,26,27,50,51,53,58,60,62,63,64,65,66,67,68,69,70,71,72,73,74,75,76,77,78,79,80] (Table 1 and Table 2). In addition, these studies have begun to explore the fundamental mechanisms through which NAD^+^ precursors exert their effects, including regulation of lipid metabolism, reduction in reactive oxidative stress (ROS), attenuation of inflammation and fibrogenesis, modulation of autophagy, and enhancement of mitochondrial function, at least in part via activation of SIRT1/SIRT3 signaling [18]. However, given the vital role that NAD^+^ plays in multiple cellular processes, these studies are likely to be the tip of the iceberg in defining the pathways that protect the liver from metabolic injury. As liver biopsies in asymptomatic healthy subjects are ethically unfeasible, the hepatic NAD^+^ levels and SIRT1 expression have only been analyzed in human liver from patients with disease and surgical controls [53,81], which limits the ability to explore the underlying mechanistic pathways involved in human MASLD.

While the preclinical evidence of the hepatoprotective effects of NAD^+^ precursors, together with biological plausibility, is encouraging, it should be noted that experimental models may differ from human MASLD in species-specific responses, diet composition, and disease induction protocols. Additionally, the doses and treatment durations used in animal studies may not directly translate to clinically relevant exposures. Therefore, caution is warranted when extrapolating these results to human populations.

To elucidate the mechanistic basis of these observations, a broad spectrum of in vitro [25,26,27,51,58,62,63,64,65,68,78,79,80] and in vivo [26,27,50,51,53,58,60,62,65,66,67,68,69,70,71,72,73,74,75,76,77] models has been instrumental to assess the therapeutic efficacy of NAD^+^-increasing interventions and to dissect the cellular and molecular mechanisms underlying their beneficial effects (Table 1 and Table 2). Within these models, NAD^+^ precursors (e.g., niacin, NAM, NR) and NAD^+^-derived metabolites have been tested, consistently demonstrating improvements in hepatic lipid handling, redox homeostasis, and inflammatory signaling.

### 4.1. Experimental In Vitro Studies

Table 1 summarizes the actions of NAD^+^-boosting strategies and related mechanisms in in vitro models of MASLD research.

#### 4.1.1. Lipid Accumulation, Lipotoxicity, and Inflammation

A broad range of in vitro studies support the positive influence of NAD^+^ precursors in attenuating hepatic steatosis, lipotoxic injury, and associated inflammatory and oxidative stress responses in hepatocyte models of MASLD/hepatic fibrosis.

Niacin significantly reduced oleic acid (OA)- and palmitic acid (PA)-induced lipid accumulation in HepG2 cells by activating the G protein-coupled receptor 109A (GPR109A)–protein kinase C (PKC)–extracellular signal-regulated kinases 1 and 2 (ERK1/2)–AMP-activated protein kinase (AMPK) signaling cascade [65] and inhibiting diacylglycerol O-acyltransferase 2 (DGAT2) activity [24], thereby lowering intracellular triglyceride levels [64]. Improved hepatic steatosis was concomitant to reduced oxidative stress, evidenced by reduced ROS generation, and inflammation, as shown by decreased interleukin (IL)-8 expression in treated cells [64].

In line with this finding, the supplementation of NR to PA-treated hepatocytes also protected cultured hepatocytes from lipid accumulation and inflammation by downregulating pro-inflammatory cytokines and upregulating adiponectin in treated cells [78]. Interestingly, reduced fat accumulation was accompanied by the overexpression of key mitochondrial targets, i.e., peroxisome proliferator-activated receptor gamma coactivator-1 alpha (PGC-1α), carnitine palmitoyltransferase 1 (CPT-1), uncoupling protein 2 (UCP2), transcription factor A, mitochondrial (TFAM), nuclear respiratory factor 1 (NRF1), and mitochondrial DNA (mtDNA). Of note, the relative expression of *Sirt1* and *Sirt3* mRNA was upregulated by NR; however, this was not accompanied by concomitant elevations in SIRT1 protein abundance and activity in conditions of PA-induced hepatic steatosis [78].

NAM treatment protected mouse liver hepatocytes (AML12, NCTC1469) and human hepatocyte cell lines (HepG2, PH5CH8) from PA-induced cell death and ER stress. This was mainly linked to induced autophagy and SIRT1 signaling in treated cells, which improved overall cellular resilience to lipotoxicity [25,63]. Similarly, NR demonstrated consistent hepatoprotective effects by reducing PA-induced lipid accumulation and inflammation in AML12, PH5CH8, and HepG2 cells [79]. Mechanistically, NR stabilized fibronectin type III domain-containing protein 5 (*Fndc5*) mRNA (which encodes IRISIN) via SIRT2-dependent deacetylation, preventing PA-mediated ubiquitination and degradation [51], while also enhancing mitochondrial biogenesis and oxidative metabolism under stress conditions. Similarly, NMN markedly alleviated PA-induced lipid accumulation and oxidative injury in NCTC1469 hepatocytes by restoring mitochondrial membrane potential and lowering ROS accumulation [62]. This effect was associated with improved ER–mitochondria coupling and enhanced mitochondrial respiration, supporting its role in preserving hepatocellular energy homeostasis. Notably, as previously reported for NAM in PA-treated HepG2 cells [68], the potential antioxidant effects of NMN may be mediated, at least in part, by the restoration of cellular NADPH pools, the primary electron donor for cytoplasmic and mitochondrial antioxidant systems [82]. Indeed, ROS elevations induced in PA-treated HepG2 cells were prevented by NAM through increased NADPH production, likely via a favorable redirection of glucose flux through the pentose phosphate pathway [68].

Preclinical evidence consistently indicates that NAD^+^ precursors, including niacin, NAM, NR, and NMN, attenuate hepatic lipid accumulation, lipotoxicity, and associated inflammatory and oxidative stress in hepatocyte models of MASLD. The most robustly supported mechanisms, observed across multiple independent studies and precursor types, include activation of SIRT-dependent signaling, induction of autophagy, enhancement of mitochondrial biogenesis and oxidative metabolism. Additional recurrent mechanisms include reduced ROS generation and decreased pro-inflammatory cytokine expression. Overall, these findings suggest that NAD^+^ precursor supplementation exerts hepatoprotective effects through convergent pathways that enhance mitochondrial function, support redox balance, and mitigate lipid-induced cellular stress.

**Table 1:** In vitro studies reporting protective effects of NAD^+^ precursors against lipid accumulation, inflammation, and fibrosis in hepatic cell models.

Target Outcome	Molecule	Cell Line/s	Experimental Details	Main Protective Effect	Reference
OA-induced steatosis, lipotoxicity	Niacin	HepG2	OA (500 µM)-induced lipid accumulation; stimulation with niacin (300 µM) for 24 h.	Activation of GPR109A resulted in remarkable inhibition of OA-induced lipid accumulation via a protein kinase *C*-extracellular signal-regulated kinase-1/2-AMP-activated protein kinase signaling pathway.	[65]
PA-induced steatosis, inflammation	Niacin	HepG2	PA (500 µM)-induced lipid accumulation; incubation with niacin (250–250 µM) for 24 h.	Inhibition of PA-induced lipid accumulation by 45–62% via inhibiting *DGAT2*; attenuation of hepatocyte ROS production accompanied by inhibition of NADPH oxidase activity; reduction in IL-8.	[64]
PA-induced steatosis	NAM	HepG2	NAM was incubated overnight (0, 1, or 2 mM, respectively) in the presence of either glucose (5 mM), PA (200 µM), or both.	Increased flux through glucose uptake, glycolysis, and pentose phosphate pathways; glucose flux redirection to the pentose phosphate pathway suggests that the favorable effect of NAM on glucose homeostasis might be linked to improved cytoplasmic redox homeostasis via increased availability of pentose phosphate pathway-derived NADPH, the main electron donor for cytoplasmic and mitochondrial antioxidant systems.	[68]
PA-induced steatosis, inflammation	NR	AML12	PA (250 µM)-induced lipid accumulation for 48 h; incubation with NR (10 µM and 10 mM) for 24 h thereafter.	Attenuation of hepatic inflammation and increased levels of mitochondrial markers.	[78]
Steatosis, inflammation	NMN	Mouse normal hepatocytes (NCTC1469)	PA (800–50 µM)-induced lipid accumulation for 36 h; followed by NMN incubation (500–0.05 μM) for 24 h.	NMN reduced PA-induced lipid accumulation and alleviated PA-induced ER stress and mitochondrial dysfunction in NCTC1469 cells, protecting against damaging stress response to mitochondrial and ER function. NMN reversed the mitochondrial membrane potential and ROS level of PA-treated cells by enhancing ER–mitochondria coupling, which controls mitochondrial respiration and energy production.	[62]
Inflammation	NR	PH5CH8 hepatocytes (immortal) and HepG2	PA (400 μM)-induced lipid accumulation in combination with NR (500 μM) for 48 h.	Reduction in PA-induced lipoinflammation and lipotoxicity seen in HepG2 cells.	[79]
PA-induced steatosis, lipotoxicity	NR	AML12 and HepG2	Cells were serum-starved for 6 h and exposed to NR (300 µM) under 150 μg/mL cycloheximide for up to 4, 8 and 16 h in a set of experiments; in another set of experiments, NR was coincubated with PA for 16 h.	Inhibition of PA-mediated ubiquitination and concomitant reduction in *Fndc5* mRNA (which encodes FNDC5, the precursor form of IRISIN) ^1^ protein in treated cells was mediated by NAD^+^-dependent SIRT2 mechanisms.	[51]
PA-induced steatosis, inflammation	NAM	AML12 and HepG2	Pretreatment with various doses of NAM (100–1000 μM) for 1 h, followed by PA (500 μM)-induced accumulation overnight.	Protection against PA-induced cell death; anti-lipotoxic properties were accompanied by autophagy induction.	[25]
PA-induced ER stress, inflammation	NAM	HepG2	Pretreatment with NAM (5 mM) for 2 h, followed by PA (200–400 μM)-induced ER stress for 16 h.	Protection against PA-induced ER stress via SIRT1 upregulation.	[63]
Fibrosis	Niacin	HSCs	Primary cultures of human HSCs were harvested from 7 cadaveric livers that were processed for transplantation (four of them had MASH with fibrosis, and 2 of them had no evidence of MASH) and incubated with pharmacologically relevant concentrations of niacin (0.25 mM and 0.5 mM) for 48 or 96 h.	Dose- and time-dependent regression of pre-existing fibrotic features in HSCs. In cells from non-MASH-fibrosis donors, niacin effectively prevented and reversed fibrosis triggered by profibrotic stimuli, including TGF-β1 and oxidative stressors. In addition, niacin markedly reduced oxidative stress responses, decreasing palmitic acid- and hydrogen peroxide-induced oxidative damage by approximately 50%.	[80]
Fibrosis	NR	Primary mouse/human HSCs	Incubation with of NR (1 mM) for 6 h and thereafter with 2 ng/mL of TGF-β1 for 24 h.	Attenuation of the activation of HSCs; downregulation of most genes important for de novo biosynthesis of NAD^+^, including the NAD^+^ salvage synthesis pathway; expression of the activation markers, i.e., *Acta2* and *Col1a1*.	[27]
Fibrosis	NR	LX-2	TGF-β1-induced fibrosis (4 ng/mL) treated with NR (500–1000 μM) for up to 96 h.	Inhibition of HSC TGF-β1-induced fibrosis activation was mediated by SIRT1 activation mechanisms and concomitant accumulations of deacetylated of SMAD2/3.	[58]
Fibrosis	NMN	Primary mouse HSCs; human HSC line LX-2 (15-PGDH knockdown LX-2 cells)	Primary mouse HSCs were treated with NMN (1000 μM) and TGF-β1 (20 ng/mL) for 24 h; LX-2 cells were treated with NMN (200–5000 μM) and TGF-β1 (20 ng/mL) for 12 h.	Suppression of HSC activation via inhibition of oxidation-mediated 15-PGDH degradation to promote prostaglandin E2 degradation.	[26]

^1^ An exerkine that attenuates autophagy and PA-induced steatosis [83]. Abbreviations: AML12, alpha mouse liver hepatocytes; AMP, adenosine monophosphate; *DGAT2*, diacylglycerol O-acyltransferase 2; ER, endoplasmic reticulum; GPR109A, G protein-coupled receptor 109A; HSCs, hepatic stellate cells; niacin, nicotinic acid; NADPH, nicotinamide adenine dinucleotide phosphate reduced form; NMN, nicotinamide mononucleotide; NR, nicotinamide riboside; OA, oleic acid; PA, palmitic acid; 15-PGDH, 15-hydroxyprostaglandin dehydrogenase; ROS, reactive oxygen species; SIRT1, sirtuin 1; SIRT2, sirtuin 2; SMAD2/3, receptor-regulated SMAD 2/3 proteins, originally identified as “suppressor of mothers against decapentaplegic”, which are intracellular transcription factors that mediate the TGF-β1 signaling pathway; TGF-β1, transforming growth factor beta 1.

**Table 2:** In vivo studies reporting the hepatoprotective effects of NAD^+^ precursors in experimental models of metabolic causes of hepatic steatosis, steatohepatitis, and fibrosis.

Target Outcome	Molecule	Animal Model	Dose/Regimen	Main Protective Effect	Reference
Hepatic steatosis	Niacin	Male Sprague-Dawley rats fed a high-fat diet (HFD).	Dietary niacin (0.5% and 1%, *w*/*w*) was given to rats for 4 wks.	Niacin prevented liver weight gain and regressed pre-existing steatosis by 42–55%, reducing liver cholesterol, and hepatic downregulated *Dgat2* expression. Significant prevention and regression of hepatic steatosis.	[60]
Hepatic steatosis	Niacin	Wildtype C57BL/6 and GPR109A-deficient (*Hcar2*–/–) mice (male, 5 wks old) were fed a HFD (60% energy from fat) firstly for 6 wks to generate a diet-induced obese model.	Oral niacin (50 mM, dissolved in water) was administered for 8–9 wks.	Inhibition of hepatic lipogenesis in C57BL/6 mice through a GPR109A-mediated signaling pathway.	[65]
Hepatic steatosis	NAM	Male rats were provided drinking water with 30% glucose or fructose ad libitum for 12 wk.	Thirty days after the beginning of carbohydrate administration, some rats were simultaneously provided NAM at 0.06% (*w*/*v*) or 0.12% (*w*/*v*) dissolved in tap water for 5 h daily over the following 8 wks.	Administration of NAM protected against sweet-beverage-induced hepatic steatosis. This protective effect was associated with reduced oxidative and inflammatory stress, downregulation of the relative abundance and activity of glucose-6-phosphate dehydrogenase, and restoration of the NADH/NAD^+^ ratio.	[66]
Hepatic steatosis	NAM	Male albino rats on a high-fat, high-fructose (HF/HF) diet for 75 d	Conventional NAM powder dose (100 mg/kg) or two doses of NAM-loaded chitosan/TPP nanoparticles (10 mg/kg and 20 mg/kg) were orally administered, as appropriate.	Treatment with NAM, either in its conventional form or encapsulated in chitosan nanoparticles, led to a significant reduction in hepatic triglyceride levels compared with the HF/HF control group, and similar decreases were observed in liver oxidative stress markers.	[67]
Hepatic steatosis	NAM	Aged, male C57BL/6J mice on HFD started at 56 wks of age	NAM was administrated via diet at doses equivalent to 37.5 and 75 mg/g/day.	Chronic NAM supplementation protected against diet-induced hepatic steatosis and enhanced glucose metabolism and hepatic redox balance in HFD-fed mice at 118 weeks of age, regardless of food intake, body weight, or body composition.	[68]
Hepatic steatosis	NR	Wildtype, C57BL/6J mice received twice-daily nicotine i.p. (0.75 mg/kg); additionally, mice were given Coca Cola^TM^ containing high-fructose corn syrup (Coke).	Twice-daily i.p. injection of NR was given in saline solution (200 mg/kg/d) for 10 wks.	Prevention of hepatic steatosis via reduced oxidative stress and prevention of mitochondrial damage by restoring protein levels of SIRT1 and PGC1α signalings.	[69]
Hepatic steatosis	NR	Apolipoprotein E-deficient mice (*Apoe*–/–) fed a HF/HS diet.	Dietary pellets containing vehicle (double-distilled water) or NR (400 mg/kg/d) for 2, 9, or 18 wks.	Prevention and reversion of hepatic steatosis by inducing a SIRT1- and SIRT3-dependent mitochondrial unfolded protein response.	[50]
Hepatic steatosis	NR	*Ldlr*–/–. Leiden mice fed a HFD.	The diet was supplemented with L-carnitine (0.4%, *w*/*w*), NR (0.3% *w*/*w*) or both (COMBI) for 21 wks.	COMBI treatment significantly attenuated HFD-induced body weight gain, fat mass gain (–17%) and hepatic steatosis (–22%); also, NR and COMBI reduced hepatic 4-hydroxynonenal adducts. COMBI reversed detrimental effects of HFD on liver metabolism.	[70]
Hepatic steatosis	NMN	Eight-week-old male, wildtype, C57BL/6J specific pathogen-free mice were randomly divided into normal diet (ND), HFD and HFD-NMN groups.	NMN (ultrapure water containing 500 mg/L (*w*/*v*) for administration via tap water for 30 wks.	Alleviation of hepatic insulin resistance and steatosis, which was linked to improved mitochondrial function and ER oxidative stress and increased hepatic content of NAD^+^; increased contact sites between ER and mitochondria were linked to increased intracellular ATP production and attenuation of lipid metabolic alterations in the livers of HFD mice.	[62]
Hepatic steatosis	NMN	Male 5 wk old db/db and C57BL/6J wildtype mice fed a regular diet.	NMN-supplemented diet (0.5%, *w*/*w*) for 4 wks.	Dietary NMN in db/db mice reduced body weight, plasma triglycerides, and hepatic triglyceride accumulation; NMN enhanced energy expenditure by increasing fat oxidation and suppressing carbohydrate oxidation; hepatic improvements included decreased fatty acid synthesis and increased β-oxidation.	[71]
Aging-related hepatic steatosis	NMN	Male Long–Evans rats (decompensated hemorrhagic model).	Oral NMN (400 mg/kg) administration.	Reduction in lactic acidosis and serum IL-6 levels, increased NAD^+^ levels, and prevention of mitochondrial dysfunction in the livers of treated mice.	[72]
Hepatic steatosis/fibrosis	Niacin	Wistar 7 wk old male rats fed a high-fat, -sucrose, and -cholesterol diet (HF/HS/HC) for 15 wks and weekly administered with an i.p. injection of low-dose CCl_4_ (400 mg/kg).	Intragastric administration of niacin (50 mg/kg/d) from the beginning of fibrosis induction.	Niacin mitigated experimental MASH/hepatic fibrosis by inhibiting the NLRP3 inflammasome/pyroptosis pathway.	[77]
Hepatic steatosis/fibrosis	NR	Hepatic steatosis induced by HFD or methionine/choline-deficient diet in wildtype and *Fndc5*–/– mice.	NR (400 mg/kg/d) for 8 wks (wildtype mice) and 12 wks (*Fndc5*–/–mice).	NR prevented body weight gain, hepatic steatosis, steatohepatitis, insulin resistance, mitochondrial dysfunction, apoptosis and fibrosis; these actions of NR were abrogated in *Fndc5*–/– mice.	[51]
Hepatic steatosis/fibrosis	NR	Wildtype and H247A dominant-negative, enzymically inactive NAMPT transgenic mice (DN-NAMPT) given normal or HFD, respectively.	NR (400 mg/kg/d) for 4 wks (as estimated according to the daily food intake).	NR prevented hepatic steatosis, steatohepatitis (i.e., reduced pro-inflammatory cytokines, Kuppfer cell accumulation and macrophage infiltration), and hepatic fibrosis.	[53]
Hepatic steatosis/fibrosis	NAM	Hepatic steatosis induced by HFD in wildtype mice.	NAM (0.25% and 1% *w*/*v*) was orally administered via tap water for 12 wks.	NR prevented body weight gain, hepatic steatosis, steatohepatitis, and hepatic fibrosis as revealed by the downregulation of specific gene markers.	[73]
Hepatic steatosis/fibrosis	Niacin	Eight-week-old male Wistar rats fed a HF HS and HC diet + low-dose CCl_4_ (i.p., 400 mg/kg, weekly).	Daily niacin administration (50 mg/kg, intragastric) for 15 wks.	Attenuation of hepatic inflammation by decreasing TNF-α and NF-κB protein levels, and inhibition of NLRP3 inflammasome activation and pyroptosis (as revealed by significant decreases in NLRP3, ASC-Caspase-1, IL-1β, IL-18); suppression of fibrosis markers (TGF-β1, α-SMA, collagen-1), thereby decreasing extracellular matrix synthesis.	[74]
Hepatic fibrosis/cirrhosis	Niacin	Male Wistar rats were treated with TAA (i.p., 200 mg/kg, 3×/wk) for 8 wks) to induce cirrhosis.	Oral niacin (50 mg/kg/d) from the beginning of cirrhosis induction.	Prevention of TAA-induced liver fibrosis, as revealed by TAA-induced reductions in circulating ALT, γ-GTP, and AP activities, preservation of hepatic glycogen, and prevention of oxidative stress by normalizing MDA and GPx levels; inhibition of hepatic collagen deposition and maintenance of liver architecture; suppression of TAA-mediated upregulation of TGF-β1, CTGF, α-SMA, MMP-2, and MMP-9.	[77]
Hepatic fibrosis	NR	Hepatic fibrosis induced by injecting i.p. CCl_4_ in mice (0.5 μL/g CCl_4_).	NR (400 mg/kg/d) orally for 8 wks.	Protection of hepatic fibrosis via increasing the activity of SIRT1 and decreasing the expression of P300, which resulted in the deacetylation of SMAD signaling in HSCs.	[58]
Hepatic fibrosis	NR	Hepatic fibrosis was induced in mice with a HF/HS/HC diet.	NR (400 mg/kg/d) was supplemented into diet and was given for 2, 4, and 7 wks.	Reduction in body weight by increasing energy expenditure, likely by upregulation of β-oxidation in skeletal muscle and brown adipose tissue; the protective effect of NR on liver fibrosis was independent of changes in liver steatosis and inflammation in obesity and liver fibrosis was unveiled by reduced collagen accumulation, and mRNA expression of fibrogenic genes in the liver.	[27]
Hepatic fibrosis	NMN	Male C57BL/6J mice injected intraperitoneally with TAA (200 mg/kg) or with 30%(*w*/*v*) CCl_4_ diluted in corn oil (2 μL/g) or vehicle (corn oil) once every two days for 32 d.	NMN (500 mg/kg) was administered by i.p. at the same time as TAA or CCl_4_ injection (32 d). Mice were sacrificed 24 h after the last TAA or CCl_4_ injection.	Inhibition of HSC activation; reduction in the production of extracellular matrix; NMN replenishment decreased PGE_2_ levels via inhibiting oxidation-mediated 15-PGDH degradation, resulting in inhibition of HSC activation to prevent liver fibrosis.	[26]
Hepatic fibrosis	NAM	Wildtype and GNMT-deficient (*Gnmt*–/–) mice on a standard diet.	At 1.5 mo. of age, NAM (50 μM) was orally administered via drinking water for 6 wks to both wildtype and GNMT-deficient mice.	Prevention of hepatic steatosis and fibrosis; reduction in hepatic SAMe content, prevention of DNA hypermethylation and normalization of the expression of critical genes involved in fatty acid metabolism, oxidative stress, inflammation, cell proliferation, and apoptosis.	[75]
Hepatic fibrosis	NAM	Male Sprague-Dawley rats were given 40% fructose dissolved in drinking water for 16 wks ad libitum.	Starting on the fifth week, drinking water containing fructose was replaced for 5 h each morning by different concentrations of NAM (5, 10 and 15 mM). After 5 h, the NAM treatments were withdrawn to continue the administration of fructose.	NAM reduced hepatic steatosis by downregulating key markers of de novo lipogenesis and mitigated liver fibrosis by lowering lipid peroxidation and preventing HSC activation.	[76]
Hepatic fibrosis	NAM	Mice were fed a CDAA-HFD.	NAM (0.5%, *w*/*v*) was orally administered via tap water for 6 wks.	NAM prevented hepatic fibrosis independent of changes in liver steatosis and inflammation.	Unpublished data *

* Unpublished data from Julia Niño-Narvión et al. (Figure S1; see also Supplementary Materials and Methods for further details). Abbreviations: α-SMA; alpha-smooth muscle actin; ALT, alanine aminotransferase; AP, alkaline phosphatase; *Apoe*, apolipoprotein E gene; ASC-Caspase-1, apoptosis-associated speck-like protein containing a CARD and cysteine-aspartic protease 1; CCL_4_: carbon tetrachloride; CDAA, choline-deficient, L-amino acid-defined diet; COMBI, combination treatment; CTGF; connective tissue growth factor; ddH20, double-distilled water; *Fndc5*, fibronectin type III domain-containing protein 5; DNA, deoxyribonucleic acid; GNMT, glycine *N*-methyltransferase; GPR109A, G protein-coupled receptor 109A; *Hcar2*, hydroxycarboxylic acid receptor 2 gene; γ-GTP, gamma-glutamyl transpeptidase; GPx, glutathione peroxidase; HFD, high-fat diet; HF/HF, high-fat, high-fructose diet; HF/HS diet, high-fat, high-sucrose diet; HF/HS/HC, high-fat, high-sucrose, high-cholesterol diet; HSCs, hepatic stellate cells; IL-6, interleukin-6; IL-18, interleukin-18; i.p., intraperitoneal; IL-1β, interleukin-1 beta; MMP-2, matrix metalloproteinase-2; MMP-9, matrix metalloproteinase-9; mRNA, messenger ribonucleic acid; NAM, nicotinamide; ND, normal diet; NF-κB, nuclear factor kappa-light-chain-enhancer of activated B cells; NMN, nicotinamide mononucleotide; NR, nicotinamide riboside; *Ldlr*, low-density lipoprotein receptor gene; P300, E1A binding protein p300; PGC1α, peroxisome proliferator-activated receptor gamma coactivator 1; 15-PGDH, 15-hydroxyprostaglandin dehydrogenase; PGE_2_, Prostaglandin E2; SAMe, S-adenosylmethionine; SIRT1, sirtuin 1; SIRT3, sirtuin 3; SMAD, receptor-regulated SMAD proteins, originally identified as “suppressor of mothers against decapentaplegic”, which are intracellular transcription factors that mediate the TGF-β1 signaling pathway; TAA, thioacetamide; TNF-α, tumor necrosis factor alpha.

#### 4.1.2. Fibrosis

In hepatic stellate cells (HSCs), NAD^+^ precursors also exhibit potent antifibrotic effects. Recent in vitro evidence demonstrated that niacin was able to reverse established collagen accumulation in HSCs derived from patients with fibrosis associated with MASH, while also preventing and attenuating collagen deposition induced by oxidative stress in non-fibrotic HSCs [80]. NR has been reported to inhibit transforming growth factor beta 1 (TGF-β1)-induced activation of primary mouse and human HSCs, leading to reduced mRNA expression of profibrogenic markers such as *Acta2* and *Col1a1* aligned with concomitant reductions in the expression of NAD^+^ biosynthetic target genes [27]. In an independent study, the protective effect of NR on TGF-β1-induced HSCs was mediated by SIRT1-dependent deacetylation of SMAD2/3 (receptor-regulated SMAD 2/3 proteins, originally identified as “suppressor of mothers against decapentaplegic”, which are intracellular transcription factors that mediate the TGF-β1 signaling pathway), leading to repression of the TGF-β1/SMAD signaling pathway [58].

In an independent study, the incubation of HSCs with NMN exerted complementary antifibrotic actions by preventing oxidation-mediated degradation of 15-hydroxyprostaglandin dehydrogenase (15-PGDH), thereby preserving prostaglandin E_2_ (PGE_2_) metabolism and attenuating HSC activation in both primary and LX-2 cells [26].

Taken together, in vitro evidence supports the notion that NAD^+^ restoration through its precursors, particularly NR and NMN, ameliorates fibrosis by limiting HSC activation, reducing oxidative stress, and modulating key profibrogenic signaling cascades. Notably, these benefits appear to be mediated primarily through SIRT1-dependent deacetylation of SMAD2/3, suppression of TGF-β1/SMAD signaling. Across independent studies, reducing oxidative stress and restoring NAD^+^ homeostasis emerge as central, consistently observed mechanisms underlying these hepatoprotective actions. While SIRT1 is a well-recognized mediator of antifibrogenic responses, direct evidence for SIRT1 regulating 15-PGDH and thereby preserving PGE_2_ metabolism is currently lacking.

### 4.2. Experimental In Vivo Studies

Table 2 summarizes the actions of NAD^+^-boosting strategies and related mechanisms in in vivo models of MASLD research. In vivo studies demonstrate that NAD^+^ precursors effectively protect against diet- and stress-induced hepatic steatosis through complementary mechanisms involving SIRT-dependent signaling, improved mitochondrial function, reducing oxidative, inflammatory stress, and preventing fibrosis.

#### 4.2.1. Hepatic Steatosis, Inflammation, and Oxidative Stress

Niacin supplementation prevents and even reverses hepatic steatosis in rats fed a HFD, confirming its lipid-lowering potential in vivo [60]. Similarly, in wildtype C57BL/6 mice, niacin inhibited hepatic lipogenesis through activation of the GPR109A-PKC-ERK1/2-AMPK signaling cascade, an effect that was abrogated in G protein-coupled receptor 109A-deficient (i.e., *Hcar2*–/–) mice [65].

The administration of NAM has also been shown to positively influence MASLD in different animal models. First, NAM prevented sweet beverage-induced hepatic lipid accumulation that was accompanied by an improved oxidative and inflammatory status in treated rats [66]. Second, NAM also protected against MASLD in mice with HFD-induced obesity by favorably influencing the hepatic gene expression of molecular lipid targets involved in lipid biosynthesis [73]. Beyond protecting against hepatic steatosis, NAM also reduced hepatic inflammation and fibrosis, as indicated by the NAM-associated downregulation of specific molecular targets [78]. Third, NAM delivered using chitosan nanoparticles was associated with improved hepatic steatosis and oxidative stress induced by a high-fat, high-fructose (HF/HF) diet in treated rats [67]. Fourth, chronic NAM supplementation prevented diet-induced hepatic steatosis while improving glucose metabolism and redox status in livers of HFD-fed mice at 118 weeks of age, independently from food intake, body weight, or body composition [68]. Fifth, NAM has also been reported to protect against fructose-induced fibrosis in rats [76].

The administration of NR displayed broad hepatoprotective actions across multiple models. In mice exposed to an HF/HF diet, NR prevented hepatic steatosis and mitochondrial dysfunction by restoring NAD^+^ content and activating the SIRT1/PGC1α axis [69]. In Western-diet-fed animals, NR supplementation both prevented and reversed hepatic steatosis through SIRT1/SIRT3-dependent induction of the mitochondrial unfolded protein response [50].

Although NR shows beneficial effects on its own, combining it with other metabolic activators can produce greater benefits. For example, in low-density lipoprotein receptor-deficient (*Ldlr*–/–) Leiden mice, co-supplementation with NR and L-carnitine reduced hepatic triglyceride accumulation, oxidative damage, and body-fat gain while normalizing liver metabolism, outperforming NR alone [70]. In another study, NR was administered as part of a broader combination of metabolic activators to male Golden Syrian hamsters fed a HFD, and this intervention significantly attenuated HFD-induced hepatic steatosis by modulating NAD^+^ metabolism, increasing hepatic levels of the free-radical scavenger ascorbate, and altering the expression of key genes involved in glutathione and ROS-related pathways, fatty acid β-oxidation, branched-chain amino acid catabolism, and the urea cycle, ultimately enhancing mitochondrial activity [84]. However, because NR was not tested alone but only as part of the combined formulation, its individual effects cannot be distinguished from those of the full combination. Nevertheless, these results provide additional evidence supporting the therapeutic potential of metabolic-activator-based interventions against MASLD [84].

Chronic NMN supplementation improved hepatic insulin resistance, hepatic steatosis, and mitochondrial dysfunction in HFD-fed mice [62]. These improvements were linked to increased hepatic NAD^+^ content, enhanced ER–mitochondria contact, and restoration of ATP production in treated mice [62]. Consistently, NMN administration also reduced mitochondrial dysfunction, along with improved lactic acidosis and systemic inflammation markers in aged or stress-related models [72]. In addition, dietary NMN supplementation in db/db mice also reduced hepatic triglyceride levels and improved hepatic fatty acid β-oxidation while suppressing lipogenesis in treated mice [85]. These effects were accompanied by reduced body weight, reflected by decreased adiposity, and elevated circulating adiponectin levels, indicating systemic metabolic improvement in NMN-treated db/db mice [71].

In summary, in vivo studies consistently demonstrate that NAD^+^ precursors attenuate hepatic steatosis, inflammation, and oxidative stress across diverse rodent models of MASLD. Mechanistically, repeated findings highlight the activation of NAD^+^-dependent signaling pathways, particularly SIRT-mediated enhancement of mitochondrial function, as central to the hepatoprotective effects. Additional shared mechanisms include reduction in ROS, improved redox balance, and downregulation of lipogenic and pro-inflammatory genes. While combination therapies may potentiate these benefits, the isolated contribution of each precursor requires further clarification. Notably, these mechanistic effects parallel findings from in vitro studies, reinforcing the relevance of NAD^+^-mediated pathways in both cellular and whole-organism contexts.

#### 4.2.2. Hepatic Fibrosis and Repair

Several NAD^+^ precursors have also demonstrated effective antifibrotic properties across chemically and metabolically induced liver injury models (Table 2).

Niacin supplementation significantly reduced inflammation by lowering the protein levels of tumor necrosis factor-alpha (TNFα) and nuclear factor kappa B (NFκB) [74]. Moreover, niacin inhibited the formation of the NLRP3- apoptosis-associated speck-like protein containing caspase recruitment domain-Caspase-1 complex, leading to decreased levels of IL-1β and IL-18 in rats fed a high-fat, -sucrose, and -cholesterol diet with CCl_4_-induced fibrosis [77]. In addition, niacin reduced TGF-β1, α-smooth muscle actin (α-SMA), and hepatic collagen-1 abundance, thereby limiting extracellular matrix synthesis. Consistently, niacin administration exhibited antioxidant properties by restoring redox balance, as reflected by normalized lipid peroxidation and GPx levels in rats treated with TAA to induce cirrhosis [77]. Niacin treatment also reduced the hepatic protein abundance of TGF-β1 and its downstream effector, the connective tissue growth factor. Additionally, niacin prevented HSC activation by suppressing α-SMA expression. Zymography assays further demonstrated that niacin attenuated the activity of matrix metalloproteinases MMP-2 and MMP-9.

NR supplementation markedly reduced liver fibrosis in both carbon tetrachloride (CCl_4_) and diet-induced fibrosis by enhancing SIRT1 activity and SMAD2/3 deacetylation, leading to repression of TGF-β1-mediated HSC activation and fibrogenic gene expression [27,73]. In line with this, oral NR also protected against MASLD as well as MASH/hepatic fibrosis in treated mice [53]. Hepatic inflammation was partially resolved by NR, as evidenced by significant reductions in the relative abundance of hepatic pro-inflammatory cytokines, Kupffer cell accumulation, and monocyte infiltration. Similarly, hepatic fibrosis was also reduced following NR treatment, as demonstrated by significant decreases in the relative levels of profibrotic markers, including α-SMA and collagen, in the livers of treated mice. Of note, NR supplementation provided greater efficacy in correcting MASLD phenotypes than SIRT1 overexpression [53].

In mice with combined hepatic steatosis and fibrosis, NR further decreased apoptosis and mitochondrial dysfunction, effects dependent on the presence of the FNDC5 [51], a precursor protein of the myokine IRISIN [83,86]. Consistently, FNDC5 plays a protective role against PA-induced hepatic steatosis by enhancing autophagy and fatty acid oxidation through the AMPK/mTOR pathway. In contrast, FNDC5 deficiency compromises these pathways, leading to disease exacerbation, while its overexpression attenuates lipid accumulation by restoring metabolic homeostasis [83].

NMN treatment also attenuated hepatic fibrosis induced by either thioacetamide or CCl_4_ administration. Such NMN-related hepatoprotective effects led to reduced extracellular-matrix production and stellate-cell activation, which was linked to inhibition of 15-PGDH oxidation and preservation of PGE_2_ metabolism in the livers of treated animals [26].

NAM also exhibited antifibrotic efficacy in models of chronic liver injury. In glycine *N*-methyltransferase (*Gnmt*) knockout mice, NAM supplementation normalized hepatic S-adenosylmethionine metabolism, a key methyl group donor, thereby reducing DNA hypermethylation, and restoring normal gene expression of molecular determinants involved in lipid oxidation, oxidative defense, and apoptosis [75]. Consistently, in an independent study using rats as an experimental model, NAM reduced hepatic steatosis by downregulating key markers of de novo lipogenesis and mitigated liver fibrosis by lowering lipid peroxidation and preventing hepatic stellate cell (HSC) activation [76].

Supporting the antifibrogenic effect of NAM, our unreported own data has also shown that NAM administration similarly prevented hepatic fibrosis in choline-deficient, L-amino acid-defined diet models (unpublished data from Niño-Narvión, J. et al., 2025) (Figure S1, see also Supplementary Materials and Methods for further details). Of note, and consistent with another study using NR instead of NAM [27], the protection against hepatic fibrosis development in treated mice was independent of changes in hepatic inflammation, as indicated by the lack of differences in the relative expression of cluster of differentiation 68 (*Cd68)* and *Il6* genes.

Taken together, these findings confirm that NAD^+^ restoration via the administration of NR, NMN, niacin, or NAM mitigates hepatic fibrogenesis and supports metabolic recovery through overlapping mechanisms involving SIRT activation, mitochondrial repair, and modulation of redox and epigenetic pathways. Beyond NAD^+^ replenishment, MNA, which is mainly produced by the liver from NAM by the enzyme NNMT [40,62], has been reported to stabilize SIRT1 protein and function and hence improve MASLD in vivo [42].

Overall, accumulating data from in vivo studies consistently demonstrate that NAD^+^ precursors attenuate hepatic fibrosis and promote liver repair across chemically and metabolically induced MASLD models. Recurrent mechanisms of action include SIRT-dependent modulation of profibrogenic signaling (notably TGF-β1/SMAD pathways), reduction in hepatic stellate cell activation, as well as enhancement of mitochondrial function and restoration of redox balance. Additional shared mechanisms involve favorable epigenetic regulation and suppression of pro-inflammatory cytokines. Importantly, many of these mechanisms align with findings from in vitro studies, supporting the translational relevance of NAD^+^-mediated hepatoprotective pathways across experimental systems and underscoring their robustness.

## 5. Human Evidence on Niacin and MASLD

Human evidence on niacin and MASLD encompasses three main exposure contexts with distinct biological and clinical implications: dietary niacin, as well as supplemental or pharmacological niacin and other vitamin B3 derivatives. Observational studies primarily assess dietary niacin and niacin equivalents derived from food sources, which generally reflect habitual intake within physiological ranges. In contrast, small interventional trials have evaluated supplementation with vitamin B3 derivatives at doses typically exceeding dietary intake but remaining within nutritional or nutraceutical ranges. Pharmacological niacin, most commonly administered as high-dose niacin, has been investigated in randomized controlled trials at gram-level doses historically used for the treatment of dyslipidemia. These substantial differences in formulation, dosage, and study design should be carefully considered when interpreting findings across studies.

Importantly, the biological effects of niacin in MASLD are strongly influenced by formulation-specific pharmacokinetics [87]. Dietary NAD^+^/NADP^+^ forms can be directly metabolized in the intestine to NAM, while niacin and tryptophan-derived niacin metabolites follow distinct absorption pathways [88]. Pharmacological preparations further diverge: immediate-release niacin formulations produce rapid systemic exposure with a shorter hepatic residence time, whereas extended-release formulations result in prolonged exposure and have been associated with a higher risk of flushing and liver enzyme elevations, which may be particularly relevant in patients with underlying liver disease [87,89].

Compared with niacin, other NAD^+^ precursors display additional metabolic differences. NAM, for example, is efficiently absorbed and does not induce flushing but may also influence hepatic methylation pathways [90]. NR and NMN appear to increase NAD^+^ levels more effectively than niacin and are generally associated with favorable tolerability profiles in humans [91,92]. These pharmacokinetic and metabolic differences likely contribute to the heterogeneous efficacy and safety outcomes observed across preclinical and clinical studies.

Beyond formulation-related variability, interpretation of human data is further complicated by concomitant pharmacological treatments frequently prescribed in MASLD populations. Many patients receive GLP-1 receptor agonists, statins, or other metabolic therapies, yet the studies included in this review did not systematically account for concurrent medication use. Moreover, potential interactions between NAD^+^ precursors and GLP-1 receptor agonist-based therapies remain insufficiently investigated. This is clearly illustrated by a recent meta-analysis [93], which reported modest effects of NAD^+^ precursors on metabolic parameters but did not evaluate interactions with GLP-1 RA therapies. Additionally, a 12-week study of NR supplementation in obese non-diabetic men showed no effect on GLP-1 secretion [94].

Taken together, variability in formulation, pharmacokinetics, and background pharmacotherapy are key factors that may partially explain the inconsistencies observed across human studies and between clinical and preclinical findings. The following sections examine epidemiological studies of dietary niacin intake and interventional studies evaluating pharmacological niacin or NAD^+^ precursors in relation to MASLD.

## 6. Epidemiological Evidence Exploring Associations Between Niacin Intake and Risk of MASLD

Evidence from human studies specifically examining the association between dietary niacin intake and MASLD is summarized in Table 3. Overall, research regarding associations between dietary niacin intake and surrogate markers of liver fat or metabolic dysfunction is limited [95,96,97,98], and findings across studies have been inconsistent, with some reporting no significant associations [99,100]. Unfortunately, two observational studies reporting a lower risk of MASLD at the moderate [98] or highest [101] ranges of dietary niacin intake could not be included in Table 3 because mean dietary niacin intake values were not eventually reported. It is worth noting, however, that in some studies, when key confounding factors, such as body mass index (BMI), physical activity, age, sex and total energy intake were considered, the associations were attenuated or no longer statistically significant [99,102]. Furthermore, studies reporting similar dietary niacin intake levels between case and control groups (mean range: 24–43 mg/d) observed inconsistent associations across cohorts.

Interestingly, the inverse relationship between dietary niacin intake and MASLD risk appears particularly evident when the participants in the study are older male adults with cardiometabolic risk factors, including obesity, smoking, alcohol consumption, and hypertension, but also in postmenopausal women and children with suspected MASLD [96,97,98].

The extent of dietary niacin intake may also be a key determinant of MASLD risk [101]. For example, in a study involving 1385 subjects with MASLD, only moderate-to-high dietary niacin intakes (ranging 22.2–26.7 mg/d) were inversely associated with MASLD risk [98]. This study reported a U-shaped relationship between dietary niacin intake and MASLD risk, with odds decreasing up to a threshold of 23.6 mg/d, above which MASLD risk was gradually increased along with dietary niacin intake higher than this value [98]. Notably, this threshold (23.6 mg/d) [98] remains well below the Tolerable Upper Intake Level of 35 mg niacin equivalents (NE)/d for adults [103], suggesting that at a dietary niacin intake above recommended dietary allowance guidelines might be protective against MASLD risk while remaining well below the Tolerable Upper Intake Level.

Beyond benefits against MASLD, dietary niacin intake has also been reported to favorably influence other advanced manifestations of this condition such as MASH and hepatic fibrosis. In this context, a higher dietary niacin intake was identified as a protective factor against potential liver fibrosis in children and adolescents with suspected MASLD [96]. In another study conducted in Chinese subjects diagnosed with sleep deficiency syndrome and MASLD, higher dietary niacin intake was reported to be directly associated with protective effects on immune function and inflammation in patients with MASLD [100]. Interestingly, this beneficial effect was linked to modulation of the gut mycobiome, characterized by an increased abundance of Rhizopus, which in turn was negatively correlated with other fungal species [99], suggesting the potential favorable influence of niacin intake in modulating gut microbiota. In a separate study, higher dietary intake of niacin was also associated with a reduced risk of all-cause mortality among individuals with MASLD [104].

In addition to population-based studies, evidence from clinical cohorts further supports the potential link between dietary niacin intake and MASLD. In a study of 148 individuals with severe obesity undergoing bariatric surgery (Figure S2, unpublished data from Martinez-Sanchez, M.A., Ramos-Molina, B. et al., 2025), MASLD was diagnosed via liver biopsy (see also Supplementary Materials and Methods for further details). Among these patients, 96 had biopsy-proven MASLD, whereas 52 did not. Dietary niacin intake was significantly lower in patients with MASLD compared to those without the disease (114.9 vs. 143.2 mg NE/day; *p*-value = 0.0358), suggesting that insufficient niacin intake may be associated with the presence of MASLD in patients with severe obesity. These findings complement population-based data and highlight the relevance of niacin intake in high-risk clinical populations.

Beyond the small number of available studies, the current observational evidence is characterized by substantial methodological heterogeneity, which may have contributed to the conflicting results. Overall, the studies differ markedly in design, population characteristics, dietary assessment methods, outcome definitions, and statistical adjustment strategies.

Several methodological limitations may hinder the interpretation of the overall evidence. First, cultural differences, largely driven by ethnic variability, may contribute to the difficulty in defining niacin intake ranges that can be considered protective against MASLD and that can be extrapolated across different populations. Second, some studies report niacin intake by tertiles or quartiles rather than as mean or median values for MASLD and non-MASLD groups, which restricts the precise estimation of intake and complicates quantitative synthesis. Third, incomplete reporting in certain studies, including the absence of detailed intake data or stratified results, further reduces transparency and limits evaluation of potential dose–response relationships. Fourth, MASLD is rarely analyzed as a primary outcome, as it is treated as a secondary endpoint, thereby limiting the ability to clearly delineate the role of dietary niacin in MASLD development or progression. Fifth, traditional dietary assessment methods present inherent limitations [105,106]. For instance, food frequency questionnaires (FFQs), dietary records, and 24-h recalls are subject to recall bias, underreporting, and intra-individual variability, which may reduce accuracy and comparability across cohorts. Sixth, the adjustment for critical confounders is a major challenge. Although most studies account for factors such as BMI, age, sex, total energy intake, alcohol consumption, and physical activity, heterogeneity in the selection and modeling of covariates may substantially influence effect estimates. Importantly, higher dietary niacin intake is frequently correlated with overall diet quality, protein intake, healthier lifestyle behaviors, and socioeconomic status. Therefore, residual or unmeasured confounding cannot be excluded. In this context, niacin intake may partly act as a proxy for broader dietary patterns associated with lower MASLD risk rather than reflecting an isolated effect of niacin itself. This limitation weakens the strength of causal inference derived from observational evidence. Seventh, heterogeneity in MASLD definitions (imaging, biomarkers, or histology) may have affected participant classification and interpretation of results. These diagnostic approaches differ markedly in sensitivity and specificity, as well as in their ability to distinguish simple steatosis from inflammatory or fibrotic stages. Imaging modalities may underestimate early inflammation or fibrosis, whereas biomarker-based definitions may reflect broader metabolic dysfunction rather than liver-specific pathology. In contrast, histological assessment remains the reference standard but is rarely feasible in large epidemiological cohorts. Consequently, individuals classified as having MASLD across studies may represent biologically distinct phenotypes and disease severities, which likely contributes to inconsistent associations observed between dietary niacin intake and MASLD risk. Finally, inter-individual genetic variability (such as polymorphisms in genes involved in lipid metabolism, e.g., *PNPLA3*, *DGAT2*), as well as inter-individual microbiome differences, may influence susceptibility to MASLD and modulate the metabolic response to dietary niacin, introducing additional layers of heterogeneity that are rarely accounted for in existing observational studies.

All these limitations underscore the need for future studies employing standardized dietary assessment methods, absolute intake reporting, consistent MASLD definitions, consideration of genetic susceptibility, and the inclusion of niacin as a primary variable. Such studies would enable more reliable comparisons and provide clearer insights into the role of niacin in MASLD pathophysiology, and these methodological advances may ultimately offer improved precision for future research. Finally, these limitations indicate that observational evidence alone cannot establish causality between niacin intake and MASLD. While epidemiological studies suggest potential associations, residual confounding and methodological heterogeneity preclude firm conclusions. Against this background, interventional studies provide a more controlled framework to directly test whether increasing NAD^+^ availability through niacin or its derivatives translates into meaningful hepatic and metabolic benefits.

## 7. Niacin Interventional Studies: Pre–Post Studies and Randomized Controlled Trials

A practical approach to enhance NAD^+^ availability in vivo involves supplementation with its biosynthetic precursors, including niacin, NAM, NR, and NMN [107,108]. Beyond efficacy signals, the clinical translation of NAD^+^-increasing strategies in MASLD requires careful evaluation of safety, tolerability, and dose–response relationships. Although vitamin B3 derivatives share the common goal of supporting NAD^+^ biosynthesis, their pharmacological properties and adverse effect profiles differ substantially according to molecular form, administered dose, and formulation.

Pharmacological doses of niacin, historically used in dyslipidemia management [109], are frequently associated with cutaneous flushing mediated by prostaglandin D_2_ release, as well as gastrointestinal discomfort. These adverse effects can reduce treatment adherence, thereby limiting the monitoring of this therapy’s efficacy [89]. In the context of MASLD, high-dose niacin has been linked to elevations in liver enzymes and, in some cases, hepatotoxicity, particularly with sustained-release formulations [89]. Additional metabolic effects, including worsening insulin resistance, have also been described [110], which may be clinically relevant in patients with obesity and T2D, thereby exacerbating cardiometabolic risk. These considerations, together with the narrow therapeutic window of niacin, limit its clinical utility.

By contrast, NAM, NR, and NMN appear safer alternatives, with no reported insulin resistance in clinical trials. However, while potential benefits of these other forms of vitamin B3 are promising, careful monitoring of metabolic effects remains warranted in clinical decision-making.

NAM, while not associated with flushing, presents distinct metabolic considerations. In contrast to niacin, NAM is widely accessible as a dietary supplement and has demonstrated safety at daily doses up to 3 g [111], though the administration of higher doses of NAM has been linked to severe, yet reversible, liver toxicity in preclinical models [112,113]. At high doses, NAM increases flux through the NNMT pathway, thereby consuming methyl donors and potentially influencing one-carbon metabolism. Sustained activation of this pathway may theoretically affect hepatic methylation capacity, homocysteine metabolism, and epigenetic regulation. Although the clinical significance of this “methylation burden” in MASLD remains unclear, it warrants attention, particularly in long-term interventions or in individuals with pre-existing metabolic dysfunction.

Other NAD^+^ precursors, such as NR and NMN, have generally demonstrated favorable safety and tolerability profiles in human trials and effectively increase circulating NAD^+^-related metabolites [91,92,114], thereby supporting their potential as safer NAD^+^-increasing strategies. However, long-term safety data in patients with chronic liver disease are still limited, and dose–response relationships have not been systematically defined. Importantly, whether increases in circulating NAD^+^ metabolites translate into meaningful hepatic NAD^+^ restoration in humans remains unresolved.

Notably, preclinical rodent studies frequently employ substantially higher weight-adjusted doses of NAD^+^ precursors than those used in human trials, complicating direct translational interpretation. For example, NR has been administered at 400 mg/kg/day in diet-induced liver fibrosis models and at 230 mg/kg/day in ApoE–/– mice, roughly corresponding to human equivalent doses of 1900 mg/day and 1100 mg/day, respectively [27,115]. Similarly, NMN has been administered to mice at 100–500 mg/kg/day, corresponding approximately to 480–2400 mg/day in humans [116,117]. By contrast, clinical trials generally use 100–1000 mg/day of NR or NMN [114]. This consistent discrepancy underscores the need for caution when extrapolating mechanistic findings from animal models to humans and highlights the importance of harmonizing dose selection across species in future translational studies.

Potential drug–nutrient interactions must also be considered. Niacin has historically been combined with statins [118,119], yet large cardiovascular outcome trials have raised concerns regarding limited additive benefit and increased adverse events in some settings. As GLP-1 receptor agonists are increasingly prescribed in subjects with MASLD and obesity, possible metabolic or hepatic interactions with NAD^+^-modulating strategies should be systematically evaluated in future trials.

Collectively, these considerations underscore that the therapeutic window for NAD^+^-boosting interventions in MASLD is likely to be molecule-specific and dose-dependent, reinforcing the need for carefully designed studies that integrate efficacy, mechanistic endpoints, and long-term safety outcomes. Within this context, interventional studies on niacin and other vitamin B3 derivatives remain relatively limited and heterogeneous. The available evidence comprises small pre–post intervention trials and a modest number of randomized controlled trials (RCTs), which differ substantially in study design, formulation, administered dose, treatment duration, and selected clinical endpoints. Moreover, study populations are heterogeneous: in some trials, MASLD was diagnosed using imaging techniques, whereas in others, diagnosis relied on metabolic or clinical criteria rather than standardized hepatic assessment. Although niacin, NAM, and other NAD^+^ precursors such as NMN and NR are considered potential modulators of hepatic NAD^+^ homeostasis, direct measurements of hepatic NAD^+^ content have not been performed in human trials, in contrast to many preclinical models. This discrepancy further complicates the extrapolation of mechanistic findings to clinical practice.

In the following subsections, pre–post intervention studies and RCTs assessing niacin and its derivatives (NAM, NMN, and NR) are shown in the following sections as preventive and therapeutic strategies to mitigate MASLD and its progression to MASH and fibrosis, highlighting both efficacy signals and limitations. These data support careful molecule-specific, dose-dependent safety monitoring in MASLD interventions.

### 7.1. Pre–Post Studies

Niacin has been the most extensively studied active form of vitamin B3 used in pre-post intervention studies (Table 4). In one such study, extended-release niacin (2 g/d) administered over 23 weeks favorably influenced hepatic and metabolic variables in 39 dyslipidemic patients; specifically, niacin treatment was associated with significant reductions in total triglycerides by −35%, liver fat by −47% (MRI-assessed), and visceral adiposity by −6% [120]. Genetic stratification in this study further revealed enhanced responses among *DGAT2* rs3060 TT homozygotes, who showed greater reductions in total triglycerides (−41.9%) and liver fat (−60.9%) compared with CC homozygotes (−20% and −25.2%, respectively), underscoring DGAT2’s role in niacin-mediated triglyceride synthesis inhibition [120]. These findings suggest a critical influence of this genetic variation in modulating the favorable response to niacin intervention in MASLD resolution. Further supporting a potential protective role by niacin against fat accumulation in the liver, dietary niacin intake was associated with improved MASLD resolution in an independent two-year lifestyle intervention study [121].

Beyond niacin, the effect of other molecular derivatives of NAM on hepatic physiology has recently been assessed (Table 4). In this regard, both short-term administration of intravenous NMN (300 mg single dose) was shown to be safe, significantly increased circulating NAD^+^ levels, and reduced triglycerides without inducing hepatic toxicity [122]. Similarly, two weeks of oral NR supplementation (500 mg twice daily) was well tolerated and produced effects comparable to those of two weeks of daily exercise (3000 m of running) performed during the preceding month, including increased plasma irisin concentrations [51], suggesting that NAD^+^ enhancement via NR may mimic certain exercise-induced metabolic adaptations. However, longer-term follow-up studies with these other safe forms of niacin are needed to elucidate their potential influence, if any, on MASLD.

### 7.2. Randomized Studies

To date, there are only a handful of RCTs evaluating niacin and other NAD^+^ precursors in people with MASLD, as summarized in Table 5.

#### 7.2.1. Niacin

Previously compiled data from RCTs have consistently shown that niacin administration, at pharmacological doses, favorably influences circulating lipid profiles, i.e., lowers total triglycerides and very-low-density lipoprotein (VLDL) triglycerides, while increasing high-density lipoprotein cholesterol (HDL-C) [119,123,124,125,126,127]. Because these lipids are key components of metabolic syndrome (MetS), and MetS itself is a well-established risk factor for cardiometabolic conditions, including MASLD [128], it has been hypothesized that niacin administration could help protect against MASLD. Specifically, an extended-release niacin formulation (Niaspan^®^) was evaluated in subjects with obesity and MASLD in the previous study. However, despite improvements in the lipid and lipoprotein profiles, niacin administration did not modify the intrahepatic triglyceride content [129]. Notably, hepatic insulin sensitivity was adversely affected in treated subjects.

Similar findings were reported in dyslipidemic male subjects with MetS in an independent study (NCT01216956) [127]. Indeed, treatment with 2 g/d of extended-release niacin for four consecutive weeks reduced total triglycerides by 28%, increased HDL-C by 17%, and decreased VLDL triglyceride production by 68%, but hepatic insulin resistance was induced, associated with hepatic diacylglycerol accumulation and impaired insulin signaling [127].

Collectively, these findings indicate that although niacin administration exerts clear beneficial effects on circulating lipid profiles [129], the treatment did not ameliorate hepatic steatosis and may, under certain conditions, contribute to hepatic lipotoxicity and insulin resistance. In contrast, acute administration of niacin has demonstrated distinct metabolic effects. This has been demonstrated in a recent mechanistic trial (NCT02808182) that assessed the impact of a single postprandial dose of 1.45–1.75 g of niacin in subjects with impaired glucose tolerance [130], whereby combined PET/CT imaging revealed increased adipose tissue fatty acid trapping and decreased hepatic and myocardial fatty acid uptake, indicating a redistribution of lipid fluxes. These findings suggest that acute niacin exposure may transiently shift substrate partitioning to reduce hepatic lipid influx [130]. One of the main discrepancies between preclinical and human findings can be attributed to several key factors, including dosing, pharmacokinetics, formulation and disease complexity. Regarding dosing, both niacin and NAM are frequently administered at high concentrations, achieving substantial hepatic exposure and providing reliable protection against steatosis, inflammation and even fibrosis in vivo [27,60,131]. In contrast, human studies use lower, clinically tolerable doses, which limit hepatic exposure and reduce the magnitude of therapeutic effects [87]. In relation to formulation and pharmacokinetics, human trials predominantly use extended-release formulations, which slow absorption and shift metabolism toward conjugative pathways [132,133]. Moreover, niacin undergoes extensive, saturable first-pass hepatic metabolism, further reducing the fraction of unmetabolized compound reaching the liver [87]. These factors together result in substantially lower effective hepatic exposure compared with the sustained high doses employed in rodent models [87].

Beyond pharmacological differences, human MASLD is typically accompanied by multiple comorbidities and heterogeneous disease stages, which are difficult to fully recapitulate in controlled preclinical models and may limit the ability of animal models to reproduce the full histopathological spectrum of the disease. Moreover, interventions in preclinical studies are often initiated at earlier disease stages than in human trials, which may contribute to the observed discrepancies in treatment effects between experimental models and patients. This complexity likely underlies the variability and more modest outcomes observed in clinical studies. In addition, several lifestyle factors, such as diet and physical activity, may modulate the metabolic impact of niacin in humans [121], further influencing therapeutic responses.

Taken together, these pharmacologic and pathophysiological differences offer a plausible explanation for why the strong preclinical effects of niacin on liver fat are not consistently observed in clinical studies.

#### 7.2.2. Other Niacin Derivatives

Other niacin derivatives, i.e., NAM and other NAM-related molecules, have also been assessed in the context of MASLD. The administration of NAM at a tolerable daily dose of 1000 mg to subjects with T2D and MASLD improved metabolic abnormalities, as shown by reduced insulin resistance and better quality-of-life measures (NCT0385088) [134]. Of note, NAM administration also reduced the serum levels of alanine aminotransferase (ALT), thereby suggesting improvement in liver function [134]. Although NAM administration was not associated with beneficial effects on liver fibrosis or steatosis in this study [134], this may be due to the relatively short follow-up period (12 weeks), which could limit the detection of favorable hepatic outcomes in NAM-treated subjects.

In this context, our research group is conducting an RCT (NICOFIB study) to evaluate the impact of NAM in subjects with liver fibrosis, in which participants will receive a daily dose of 1.2 g/m^2^ NAM for one year (NCT06599918), potentially overcoming the limitations of the previous study.

In addition to NAM, other NAD^+^ precursors have also been clinically tested. A double-blind, placebo-controlled, three-arm RCT evaluated the combination of NR and pterostilbene (NRPT) in 111 adults with MASLD (NCT03513523) [135]. Participants received either placebo, a moderate dose (250 mg NR + 50 mg pterostilbene (PT); NRPT-1 group), or a high dose (500 mg NR + 100 mg PT; NRPT-2 group) for 12 weeks. No significant changes were observed in hepatic fat content in any of the treated groups; however, NRPT supplementation was well tolerated. Notably, in the NRPT-1 group, serum ALT levels and the saturated fatty acid ceramide C14:0 were reduced after the intervention, suggesting potential therapeutic effects through modulation of hepatic inflammation [135].

Taken together, these human studies indicate that NAM and its related derivatives can positively modulate systemic metabolic homeostasis and inflammation; however, whether these effects translate into measurable hepatic improvements in patients with MASLD is still uncertain. It also remains to be determined if their actions are primarily mediated through their role as NAD^+^ precursors or other mechanisms. Further investigation is needed to establish optimal dosing, treatment duration, and patient selection to clarify their potential therapeutic role in MASLD.

**Table 5:** Randomized clinical trials reporting the hepatic effects of NAD^+^ precursors in human cohorts.

Molecule/Dose Regimen	Population Characteristics	Dose, Duration of Treatment, and Main Clinical Characteristics	Primary Outcomes	Main Results	Adverse Effects	NCT	Reference
Oral niacin	27 subjects with obesity and MASLD * with a mean age 42 (2) y.	Niacin (Niaspan) 500–2000 mg/day over 16 wks. Participants on niacin underwent eight biweekly clinic visits over 16 wk, with adherence monitored through pill counts at each visit, weekly nurse contacts, and biweekly phone calls.	IHTG	Niaspan reduced plasma TG, VLDL-TG and VLDL-ApoB concentrations by lowering VLDL-TG and VLDL-ApoB secretion but had no effect on IHTG content.	No adverse events were reported in the study.	n.a.	[129]
Oral niacin	20 subjects with IGT and 19 subjects with NGT.	Oral niacin: 100 mg at 0 min, then 150 mg every 30 min (A1: 8 doses, total 1.45 g; B1: 10 doses, total 1.75 g) to sustain suppression of intracellular lipolysis for ≥4 h. Participants underwent two randomized, standardized 6 h postprandial protocols with or without oral niacin, following a 906 kcal liquid meal; niacin was administered as 100 mg at time 0 and repeated 150 mg doses every 30 min up to 330 min, for total doses of 1.45–1.75 g, while organ-specific fatty acid fluxes were quantified using PET/CT and stable isotopic tracers.	Postprandial distribution of DFA between adipose tissue and lean organs (liver and heart)	DFA partitioning with reciprocal reduction in liver and in muscle. Niacin also robustly reduced cardiac and liver total (DFA + NEFA) postprandial fatty acid uptake. Short-acting niacin administered postprandially thus enhances adipose tissue DFA trapping and markedly reduces postprandial hepatic and cardiac fatty acid uptake.	No adverse events were reported in the study.	NCT02808182	[130]
Oral NR and pterostilbene	111 adult participants with MASLD * with a mean age of 52.1 (11.7) y (placebo group), 56.5 (9.0) y (NRPT-1) and 54.1 (8.3) y (NRPT-2)	Participants were distributed into 3 groups: the placebo group (receiving four capsules); The NR and pterostilbene (NRPT) 1 group (receiving two NRPT capsules, containing 250 mg NR + 50 mg PT) and two placebo capsules; and the NRPT 2 group (receiving four NRPT capsules, containing 500 mg NR + 100 mg PT). Duration: 6 mo. Participants attended five in-clinic visits, screening (up to 4 wk before baseline), baseline/randomization (on day 0), Weeks 6 and 12, and end of study (on wk 26, day 180), with a telephone follow-up 2 wks later; investigational product accountability was ensured through on-site visits and investigator diary review.	Hepatic fat fraction	No significant change was seen in the primary endpoint of hepatic fat fraction with respect to placebo. NRPT at the recommended dose is safe and may hold promise in lowering markers of hepatic inflammation in patients with MASLD *.	A total of 41 participants (36.9%) experienced 87 treatment-emergent adverse events, mostly mild gastrointestinal symptoms or abnormalities in blood tests. No severe adverse events occurred. Treatment-related adverse events were reported in 21 cases: 11 in placebo, 4 in the NRPT 1× group, and 6 in the NRPT 2× group.	NCT03513523	[135]
Oral niacin	20 non-diabetic, dyslipidemic male participants with metabolic syndrome.	Each participant received 2 g per day of extended-release niacin (Niaspan^®^) or placebo for 4 wks, with a 4 wk washout period between treatments. The primary interventions were oral administration of Niaspan or placebo for 8 wks. Participants attended a total of four clinic visits, corresponding to the beginning and end of each treatment period. Monitoring included assessments of plasma lipid profiles at the beginning and end of each treatment period.	Insulin sensitivity assessed by the two-step euglycemic-hyperinsulinemic clamp, together with VLDL–triglyceride production rate	Niaspan (2 g/d over 8 wk) in 20 dyslipidemic men reduced triglycerides by 28%, increased HDL-C by 17%, and lowered VLDL-TG production by 68%, but induced hepatic insulin resistance, associated with diacylglycerol accumulation and reduced insulin signaling in hepatocytes.		NCT01216956	[127]
Oral NAM	70 diabetic MASLD * patients (61 with follow-up).	NAM 1000 mg daily over 12 wks with weekly phone calls and monthly visits to assess adherence.	Liver steatosis scores	NAM at a dose of 1000 mg daily was tolerable, improving metabolic abnormalities and quality of life, with no effect on liver fibrosis or steatosis.	No adverse events in the placebo group. In the NAM group, two cases of nausea/vomiting and one of fatigue occurred in week 1; all were mild, self-limited, and required no intervention or discontinuation. No liver enzyme elevations were observed.	NCT0385088	[134]

Where applicable, data are expressed as mean (standard deviation, SD) unless otherwise specified. Abbreviations: ApoB, apolipoprotein B; CT, computed tomography; DFA, dietary fatty acid; HDL-C, high-density lipoprotein cholesterol; IHTG, intrahepatic triglyceride content; IGT, impaired glucose tolerance; MASLD: metabolic dysfunction-associated steatotic liver disease; mo., month; n.a., not available; NAM, nicotinamide; NCT, National Clinical Trial; NEFA, non-esterified fatty acid; NGT, normal glucose tolerance; NR, nicotinamide riboside; NRPT, nicotinamide riboside and pterostilbene; PET, positron emission tomography; PET/CT, positron emission tomography/computed tomography; PT, pterostilbene; TG, triglycerides; VLDL, very-low-density lipoprotein; VLDL-TG, VLDL triglyceride; VLDL-apoB, VLDL apolipoprotein B; wk, week; wks, weeks. * When indicated, the current term MASLD that appears in this table was used instead of the former term nonalcoholic fatty liver disease (NAFLD).

## 8. Conclusions

Current evidence supports a central role for NAD^+^ metabolism in the pathophysiology of MASLD. However, it is important to note that most of the mechanistic and therapeutic insights available to date arise from preclinical studies, whereas evidence from human trials remains limited and inconsistent. Restoration of hepatic NAD^+^ levels through dietary or pharmacological precursors, such as niacin, NAM and NAM-related derivatives, has consistently demonstrated hepatoprotective effects in preclinical models of MASLD. These benefits have been primarily mediated by the rewiring of mitochondrial function and attenuation of lipotoxic and inflammatory responses. While the underlying mechanisms are complex and require further investigation, the activation of sirtuin signaling strongly emerges as a key contributor to hepatic metabolic improvement in experimental settings. Nevertheless, translating these findings into clinical benefit remains challenging, as human trials have so far yielded inconclusive results, likely due to heterogeneity in type, dosage, treatment duration, and patient characteristics. Moreover, direct measurement of hepatic NAD^+^ content in human studies is largely lacking, further complicating mechanistic interpretation. Safety signals, including hepatotoxicity with high-dose niacin and potential methylation burden with NAM, require careful consideration. In summary, while NAD^+^-increasing strategies hold promise, current human data do not yet provide consistent support for their therapeutic efficacy in MASLD. Key gaps persist regarding optimal dosing, treatment duration, patient selection, and long-term safety. Addressing these gaps will be essential to inform the design of future clinical trials and dietary or pharmacological interventions.

## 9. Perspectives and Future Directions

Future research should aim to bridge the translational gap between experimental and clinical findings on NAD^+^-increasing strategies in MASLD. Current evidence highlights the potential of niacin, NAM, NR, and NMN to mitigate hepatic steatosis, inflammation and fibrosis in experimental models; however, heterogeneity in study design, dosage, and endpoints in human trials limit definitive conclusions. To address these gaps, future research should focus on the following key areas:

### 9.1. Biomarker-Guided Dosing and Patient Stratification

Well-powered RCTs are needed to determine the optimal form, dosage, and duration of NAD^+^ precursor supplementation in MASLD patients. Future studies should also explore interactions with dietary interventions and concomitant medications [136,137]. Mechanistic studies should also clarify how NAD^+^ metabolism intersects with mitochondrial homeostasis, immune signaling, and hepatic regeneration [138,139]. Biomarkers, including circulating vitamin B3-derived metabolites, sirtuin activity, or mitochondrial function indices, could help tailor dosing to individual metabolic or hepatic profiles. Host-specific factors, such as baseline metabolic phenotype, comorbidities, or gut microbiome composition, may influence responsiveness.

### 9.2. Standardized Clinical Trial Design

Trials should employ consistent MASLD diagnostic criteria and endpoints, ideally combining imaging-based hepatic fat quantification with metabolic and biochemical readouts. Integration with lifestyle interventions and concomitant medications (e.g., statins or GLP-1 receptor agonists) should also be systematically evaluated to understand potential interactions and maximize translational relevance.

### 9.3. Long-Term Safety and Tolerability

Safety assessments should focus on hepatic effects, flushing, and NNMT-mediated methylation burden associated with NAM. Evaluations should include dose-dependent safety profiles and potential drug–nutrient interactions. While hepatotoxicity and flushing are well-documented with high-dose niacin, NAM-related adverse effects have generally been observed only at high doses. These studies are critical to support broader clinical adoption of NAD^+^-based interventions.

### 9.4. Mechanistic Investigations

Experimental and translational studies should explore how NAD^+^ metabolism intersects with mitochondrial homeostasis, immune signaling, hepatic regeneration, and fibrogenic pathways. Understanding these mechanisms will clarify how NAD^+^ restoration contributes to hepatoprotection and whether specific precursors offer advantages in distinct disease stages.

### 9.5. Gut–Liver Axis and Microbiome Interactions

The interplay between NAD^+^ metabolism and the gut microbiome should also be explored in future studies [140]. The gut microbiome can influence NAD^+^ availability via microbial synthesis (Preiss–Handler pathway) [141,142] and modulation of hepatic metabolism through microbial metabolites such as short-chain fatty acids, bile acids, ethanol, or TMAO [143]. NAD^+^ precursors may also reshape the gut microbiota, improve intestinal barrier function, and reduce systemic and hepatic inflammation [140]. Multi-omics approaches (metagenomics, metabolomics, transcriptomics) will be essential to uncover bidirectional interactions and identify patient-specific determinants of response.

### 9.6. Broader Translational Applications

Emerging preclinical data also suggest that NAD^+^ restoration may protect against hepatic injury beyond MASLD, including alcohol-induced liver injury, drug-induced hepatotoxicity, and senescence- or fibrosis-associated damage [144,145,146,147,148,149,150,151] (Table S1). Notably, NR and NMN have shown hepatoprotective effects through SIRT1/PGC1α and SIRT3/NRF2 pathways [144,145,146], while NAM may promote hepatocyte regeneration in vivo [147]. These findings highlight the therapeutic versatility of NAD^+^-modulating strategies and justify exploration in diverse hepatic disorders.

### 9.7. Sex-Specific Considerations

It should also be noted that most preclinical studies have been conducted in male animals, whereas human studies include both sexes without specific subgroup analyses. The impact of sex on NAD^+^ metabolism and MASLD progression or response to therapy remains unclear. Future studies should incorporate sex as a biological variable to determine whether NAD^+^-based interventions exert differential effects in males and females, which may inform precision therapy approaches.

Collectively, focusing on these research priorities will be critical to translate the promising preclinical evidence into safe, effective, and personalized NAD^+^-based interventions for MASLD and potentially other liver diseases.

## Figures and Tables

**Table 3:** Epidemiological (cross-sectional, cohort and case–control) studies on the hepatic effects of estimated dietary niacin intakes.

Dietary Niacin Intake	Population Characteristics	Dietary Assessment	Energy Intake Adjustment	Main Results	Reference
Niacin: 19.2 mg/d ^†^	N = 1385 subjects with MASLD, aged between 18 and ≥60 y	Dietary assessment was performed using two 24 h dietary recalls.	No	A U-shaped relationship was observed between dietary niacin intake and MASLD risk, with an inflection point at 23.6 mg/d, suggesting that moderate intake may reduce MASLD prevalence.	[98]
Niacin (mg/d): 27.2 (5.16) NE (mg/d): 43.3 (6.9)	N = 222 subjects with MASLD (55.2 ± 11.9 y)	Dietary assessment was conducted using a 101-item Spanish FFQ.	Yes	Dietary niacin intake did not differ between subjects with or without MASLD, and fully adjusted models showed no direct association. However, smoking, female sex, and higher BMI were consistently linked to MASLD, and niacin intake <35 mg/d was associated with lower risk in non-linear analyses.	[99]
Niacin (mg/d) from ≤16.3 to ≥29.2 ^†^	N = 4378 subjects with MASLD * (mean age 52.7 ± 19.3 y)	Dietary intake was assessed using two 24 h dietary recalls.	Yes	Niacin intake (>22.2 mg/d) across four quartiles was associated with a lower risk of MASLD * (ORs for Q2–Q4 vs. Q1 = 0.84, 0.80, 0.69; *p*-value = 0.001), with variation by hypertension status (*p*-value = 0.033).	[101]
Niacin (mg/d): 35.16 (84.37)	N = 435 postmenopausal women with MASLD *, aged 18–80 y	Dietary intake was assessed using two 24 h dietary recalls.	No	Higher dietary niacin intake showed a nonlinear association with MASLD * risk in postmenopausal women, with odds peaking at 15.8 mg/d (OR 1.04, 95% CI 1.02–1.07) and declining to a plateau at ~40 mg/d (restricted cubic splines), though no significant association was found in fully adjusted models.	[97]
Niacin (mg/d): 16.21 (0.85)	N = 138 children and adolescents with MASLD * aged 10–18 y.	Dietary assessment was performed using one 24 h dietary recall.	No	Higher dietary niacin intake was identified as an independent protective factor against potential liver fibrosis in children with suspected MASLD *, with OR 0.862 (*p*-value = 0.019).	[96]
MASLD_NP * Niacin (mg/d): 11.22 (2.56); MASLD_P * Niacin (mg/d): 10. 98 (2.52)	N = 245 MASLD * subjects, 106 cases of spleen deficiency syndrome (MASLD_NP *) and 139 cases without spleen deficiency syndrome (MASLD_P *)	Dietary assessment was performed using a Chinese 81-item FFQ.	Yes	Among MASLD * patients, niacin intake was negatively correlated with the abundance of Rhizopus (r = −0.39, *p*-value = 0.025), suggesting that higher niacin intake may be associated with a potentially beneficial modulation of gut fungi and a protective effect on immune function and inflammation in MASLD * patients with spleen deficiency syndrome.	[100]
Liver steatosis Niacin (mg/d): 24.32 (13.27); liver fibrosis Niacin (mg/d): 24.47 (12.63)	N = 2458 Subjects with liver steatosis with a mean age 53.74 (16.29) and 655 with hepatic fibrosis with a mean age 55.64 (15.84)	Dietary assessment was performed using two 24 h dietary recalls.	No ^#^	Niacin intake was not significantly associated with either liver steatosis or fibrosis. Compared with the lowest tertile, higher niacin consumption did not show a protective or adverse effect on steatosis (fully adjusted OR for T2 vs. T1 = 1.25, 95% CI: 0.89–1.76; T3 vs. T1 = 1.22, 95% CI: 0.77–1.92). Similarly, no significant associations were observed for liver fibrosis (OR for T2 vs. T1 = 0.98, 95% CI: 0.55–1.76; T3 vs. T1 = 1.12, 95% CI: 0.74–1.70).	[95]
Niacin (mg/d): 28.3 (9.7)	N = 295 MASLD * subjects with a mean age of 43.5 (14.5) y	Dietary intake was evaluated using a 168-item Iranian FFQ.	Yes	The Index of Nutritional Quality (INQ) of niacin was significantly lower in MASLD * patients compared to controls (1.33 vs. 1.42; *p*-value < 0.0001). Logistic regression showed an inverse association in the age- and sex-adjusted model (OR = 0.26; 95% CI: 0.15–0.46; *p*-value < 0.0001), but the association was attenuated and not significant after full adjustment (OR = 0.69; 95% CI: 0.35–1.36; *p*-value = 0.28).	[102]
Niacin (mg/d): 131.3 (91.9)	N = 148 Individuals with severe obesity undergoing bariatric surgery.	Dietary intake was evaluated using a 136-item Spanish FFQ.	No	Dietary intake of niacin levels was significantly lower in biopsy-proven MASLD patients (N = 96) compared to subjects without MASLD (N = 52): 114.9 vs. 143.2; *p*-value = 0.0358.	Unpublished data ^‡^

Where applicable, data are expressed as mean (standard deviation, SD) unless otherwise specified. Abbreviations: CI, confidence interval; 24 h dietary recall, 24 h dietary recall; FFQ, food frequency questionnaires; INQ, Index of Nutritional Quality; MASLD, metabolic dysfunction-associated steatotic liver disease; MASLD_NP, metabolic dysfunction-associated steatotic liver disease with no spleen deficiency syndrome; MASLD_P, metabolic dysfunction-associated steatotic liver disease with spleen deficiency syndrome; N, number of participants; NE, niacin equivalents; OR, odds ratio; Q1–Q4, quartile 1–quartile 4; T1–T3, tertile 1–tertile 3; y, years. * When indicated, the current term MASLD that appears in this table was used instead of the former term nonalcoholic fatty liver disease (NAFLD). ^†^ Dietary niacin intake was reported as a categorical variable in the original studies. For studies that did not provide intake data as mean (SD), the corresponding authors were contacted on at least two occasions to request this information; however, no responses were received. ^#^ Dietary niacin intake was adjusted to total cholesterol intake. ^‡^ Unpublished data from Martinez-Sanchez, M.A., Ramos-Molina, B. et al., 2025 (Figure S2; see also Supplementary Materials and Methods).

**Table 4:** Pre–post interventional studies assessing the hepatic effects of NAD^+^ precursors in human cohorts.

Molecule/Dose Regimen	Population Characteristics	Dose, Duration of Treatment, and Main Clinical Characteristics	Main Results	NCT	Reference
Oral niacin	N = 39 participants with dyslipidemia and at least one metabolic or cardiovascular risk factor, including MASLD *, with a mean age of 55.10 (9.10) y.	Participants with previous diagnosis of MASLD * or central obesity received extended-release niacin (Niaspan^®^) once daily at bedtime, with doses titrated from 375 mg to 2000 mg over the study period (23 wk intervention).	Niacin treatment significantly increased HDL-C and decreased triglycerides, total cholesterol, several apolipoproteins, liver fat content (–47.2 [32.8%]), and visceral adipose tissue (–6.3 [15.8%]), reducing the proportion of participants with hepatic steatosis to 48.7%; liver enzymes showed modest reductions, and body weight decreased slightly (–1.17 [2.44] kg); greater liver fat and visceral fat reductions were observed in participants with weight loss ≥1 kg, although liver fat decreased even in those who gained weight.	n.a.	[120]
Dietary niacin	N = 202 adults of whom 58 had MASLD * at baseline with an average age of 45 (38.5) y.	A 2 y lifestyle intervention combined individualized dietary counseling and physical activity guidance. Counseling sessions were monthly for the first 6 mo. and every 3 mo. thereafter, focusing on ≥5% weight loss, reduced total and saturated fat, increased fiber, fruits, and vegetables, and limited alcohol.	During the lifestyle intervention, MASLD * was resolved in 23 participants. A one-standard-deviation increase in dietary niacin intake was associated with higher odds of MASLD * resolution (OR 1.77, 95% CI: 1.00–3.43), suggesting that high niacin intake may favor the reduction in liver fat.	n.a.	[121]
Intravenous NMN	N = 10 Healthy participants with an average age of 43.4 (12.6) y without abnormalities or general disorders, urinalysis, electrocardiograms, and chest radiographs	Each participant received a single intravenous infusion of 300 mg NMN dissolved in 100 mL saline, administered at a rate of 5 mL/min. After 1–2 mo., samples were analyzed.	Intravenous NMN administration is safe and beneficial in humans, significantly increasing blood NAD^+^ levels and reducing triglycerides without affecting liver enzymes or causing hepatic damage.	n.a.	[122]
Oral NR	N = 6 healthy volunteers ^‡^	GMP-grade NR (NIAGEN^®^; 500 mg twice daily) for two weeks. Blood samples (100 µL) were collected from the fingertip before and after the two-week supplementation. One month earlier, the same participants had performed a 2 wk physical exercise intervention consisting of running 3000 m/d.	Both two weeks of physical exercise and two weeks of supplementation with GMP-grade nicotinamide riboside (NIAGEN^®^; 500 mg twice daily) significantly increased plasma irisin concentrations.	n.a.	[51]

Abbreviations: CI, confidence interval; GMP, good manufacturing practice; HDL-C, high-density lipoprotein cholesterol; mg, milligram; MASLD: metabolic dysfunction-associated steatotic liver disease; n.a., not available; NAD^+^, nicotinamide adenine dinucleotide (oxidized form); NMN, nicotinamide mononucleotide; NR, nicotinamide riboside; m/d, meters/day; mo., months; OR, odds ratio; wk, week; wks, weeks; y, years. * When indicated, the current term MASLD that appears in this table was used instead of the former term nonalcoholic fatty liver disease (NAFLD). ^‡^ Characteristics from healthy volunteer subjects were not reported in the study.

## Data Availability

Proposals relating to the data access should be directed to the corresponding authors. To gain access, data requestors will need to sign a data access agreement.

## Supplementary Materials

The following supporting information can be downloaded at https://www.mdpi.com/article/10.3390/nu18060996/s1. Table S1: In vitro and in vivo studies reporting the hepatoprotective effects of NAD^+^ precursors in experimental models of alcohol-induced hepatic steatosis, liver regeneration, drug-induced liver injury, and hepatocellular carcinoma; Figure S1: Effect of NAM treatment on hepatic fibrosis in male wildtype mice fed a choline-deficient, L-amino acid-defined (CDAA) high-fat diet (CDAA-HFD); Figure S2: Dietary niacin intake in subjects with morbid obesity; Supplementary Materials and Methods. References [144,145,146,147,148,149,150,151,152,153,154,155] are cited in the Supplementary Materials.

## Abbreviations

The following abbreviations are used in this manuscript:

ALT	Alanine aminotransferase
AML12	Alpha mouse liver hepatocytes
CCl_4_	Carbon tetrachloride
CD38	Cluster of differentiation 38
CDAA-HFD	Choline-deficient, L-amino acid-defined high-fat diet
*DGAT2*	Diacylglycerol O-Acyltransferase 2
ER	Endoplasmic reticulum
FNCD5	Fibronectin type III domain-containing protein 5
GNMT	Glycine N-methyltransferase
GPR109A	G protein-coupled receptor 109A
HDL-C	High-density lipoprotein cholesterol
HFD	High-fat diet
HSCs	Hepatic stellate cells
MNA	1-methylnicotinamide
NAD^+^	Nicotinamide adenine dinucleotide (oxidized form)
NADH	Nicotinamide adenine dinucleotide (reduced form)
NAM	Nicotinamide
NAMPT	Nicotinamide phosphoribosyltransferase
NMN	Nicotinamide mononucleotide
NNMT	Nicotinamide riboside kinase
NR	Nicotinamide riboside
15-PGDH	15-hydroxyprostaglandin dehydrogenase
PGE_2_	Prostaglandin E2
ROS	Reactive oxygen species
SIRTs	Sirtuins
α-SMA	Alpha-smooth muscle actin
SMAD	Receptor-regulated SMAD 2/3 proteins, originally identified as “suppressor of mothers against decapentaplegic”, which are intracellular transcription factors that mediate the TGF-β signaling pathway
TMAO	Trimethylamine N-oxide (TMAO)
T2D	Type 2 diabetes mellitus
TGF-β1	Transforming growth factor beta 1
VLDL	Very-low-density lipoprotein
