# Supplementary material for: Niacin Derivatives in MASLD: Metabolic and Therapeutic Insights
Source: Nutrients. 2026 Mar 20;18(6):996. doi: 10.3390/nu18060996 (PMC13029165; doi:10.3390/nu18060996)
Supplement: Supplementary file 1 [file nutrients-18-00996-s001.zip › nutrients-4160930-supplementary.pdf]

nutrients MDPI

Supplementary Material

Niacin Derivatives in MASLD: Metabolic and Therapeutic Insights

Table S1. In vitro and in vivo studies reporting the hepatoprotective effects of NAD⁺ precursors in experimental models of alcohol-induced hepatic steatosis, liver regeneration, drug-induced liver injury, and hepatocellular carcinoma.

Target outcome	Molecule	Animal model	Dose/regimen	Main protective effect	Reference
In vitro					
Hepatocellular carcinoma	NR	HepG2	Cells were cultured in the FBS-free medium containing TGF-β1 (4 ng/mL) and NR (1 mM) for 48 h.	Inhibition of TGF-β1-induced migration and invasion of HepG2 cells.	[148]
Liver regeneration	NR	Primary hepatocytes	NR (500 µM/d) [1] for 2 wks.	Increased oxygen consumption and glucose output in cultured hepatocytes.	[149]
In vivo					
Alcohol-induced hepatic steatosis	Niacin	C57BL/6J mice treated with Lieber-DeCarli ethanol liquid diet	Dietary niacin (0.5%, w/v) was given for 4 wks.	Niacin supplementation attenuated liver injury induced by chronic alcohol exposure, which was associated with alleviated hepatic lipid peroxidation and increased liver GSH concentrations.	[150]
Alcohol-induced hepatic steatosis	Niacin	Isocaloric liquid diets: control, ethanol (EtOH)	Dietary supplementation of niacin (750 mg/L) for 8 wks.	Prevention of alcoholic steatosis; enhanced hepatic fatty acid oxidation and reduced hepatic de novo lipogenesis.	[151]
Alcohol-induced hepatic steatosis	NR	C57BL/6J mice treated with Lieber-DeCarli ethanol liquid diet	NR (400 mg/kg/d) orally (o.g.) for 16 d.	Protection against ethanol induced liver injuries via replenishing NAD+; reduction of oxidative stress, and activation SIRT1-PGC-1α-mitochondrial biosynthesis.	[145]
Alcohol-induced hepatic steatosis	NMN	Male C57BL/6J mice fed a 5% ethanol liquid diet	Mice were acclimated with control liquid diet for 5 days, then fed a 5% ethanol liquid diet for 10 days. On day	Protection against alcoholic liver injury in a mouse model, potentially through the upregulation of the cellular NAD⁺-SIRT1 pathway.	[144]

			16, ethanol-fed groups received a single ethanol gavage (5 g/kg). NMN (300 or 500 mg/kg) was administered daily i.p. for one year [2].		
Oxidative stress liver damage	NMN	C57BL/6 wild-type mice and *Sirt3*-deficient mouse; for acetaminophen treatment to at young (8 wk) or aged (96 wk) mice	NMN (500 mg/kg) was administered i.p. for 4 wks every other day for 4 wks and then sacrificed after 24 h of the last injection.	Aged mice treated with NMN improved stress resistance against acetaminophen (APAP)-induced liver injury, indicating that NMN restored NRF2-mediated adaptive oxidative stress homeostasis via the SIRT3-NRF2 axis.	[146]
Liver regeneration	NAM	Male C57BL/6 mice subjected to PH	For the study group, NAM (250 mg/kg/d) was administered i.p. 6 h prior to PH and once daily subsequently for a total of 3 d.	NAM supplementation promoted the proliferation of hepatocytes and accelerated the recovery of liver tissue; SIRT1 upregulation following treatment with NAM, suggesting that NAM may promote liver regeneration through activation of SIRT1.	[147]

Abbreviations: APAP, acetaminophen; EtOH, ethanol; FBS, fetal bovine serum; GSH, glutathione; i.p., intraperitoneal; mM, milimolar; μM, micromolar; NAM, nicotinamide; NAD+, nicotinamide adenine dinucleotide (oxidized form); NMN, nicotinamide mononucleotide; NR, nicotinamide riboside; NRF2, nuclear factor erythroid 2–related factor 2; o.g., oral gavage; PH, partial hepatectomy; PGC-1α, peroxisome proliferator-activated receptor gamma coactivator 1-alpha; SIRT1, sirtuin 1; SIRT3, sirtuin 3; TGF-β1, transforming growth factor beta 1; wks, weeks; d, days; w/v, weight/volume; y, year.

[1] which we have found to be optimal for increasing NAD+ content.

[2] Dose selection was based on previous safety studies showing no toxicity at 300 mg/kg/day for 1 y.

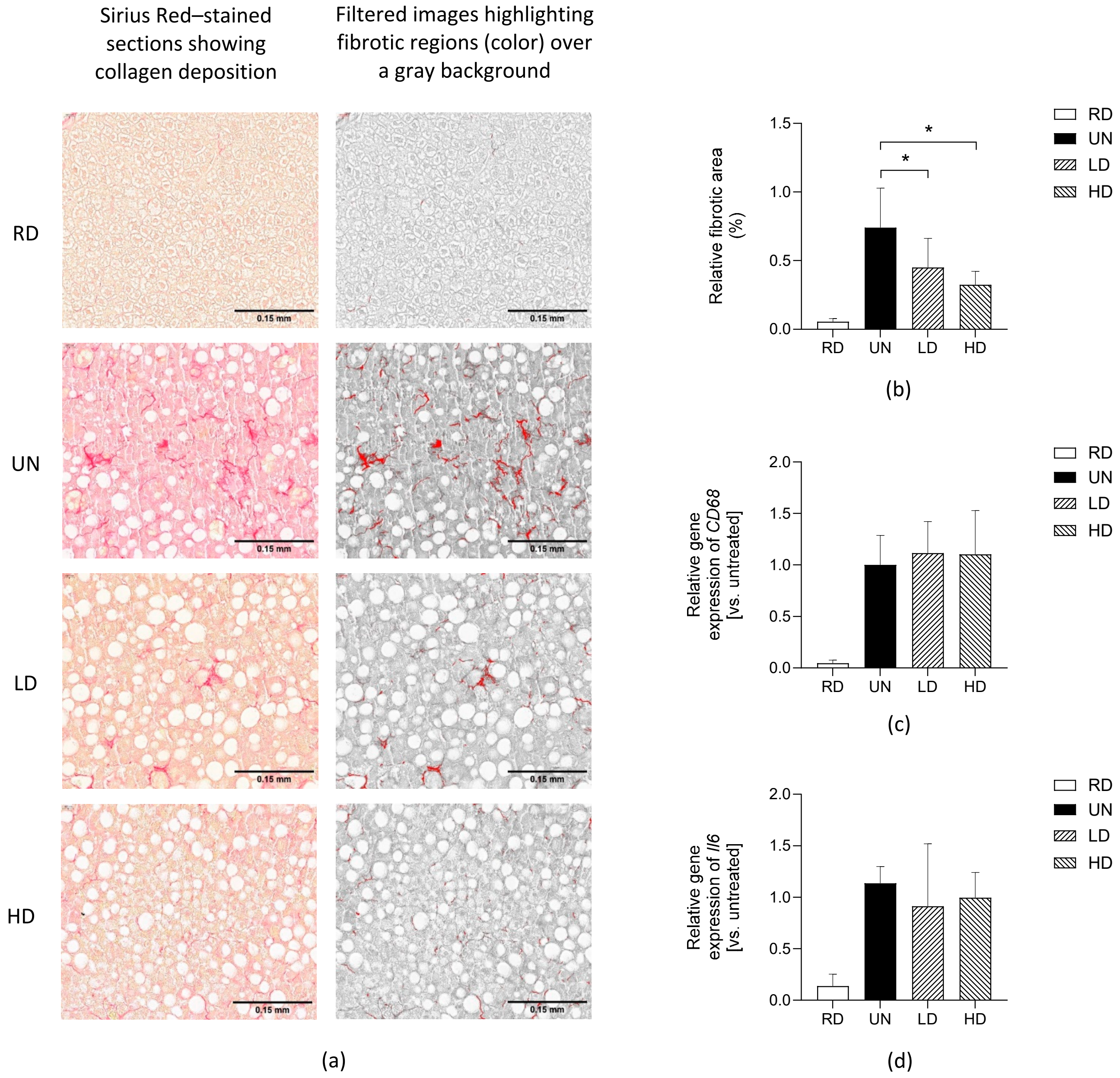

Figure S1. Effect of NAM treatment on hepatic fibrosis in male wild-type mice fed a choline-deficient, L-amino acid-defined (CDAA) high-fat diet (CDAA-HFD). Data were expressed as mean, standard deviation (n = 5-8 mice/group). (a) Representative Sirius Red–stained liver sections from the different experimental groups. The left column shows original histological images, while the right column displays the same images after applying a digital filter to isolate and highlight fibrotic regions (colored) from non-fibrotic tissue (gray background) Scale bar = 150 µm. Images on the left side of the panel show the sections as observed under the microscope, while images on the right side highlight the specific fibrogenic signal. (b) Relative fibrotic area (%) in liver specimens. Quantification was performed on ten randomly selected fields per section using Python-based image analysis (c) Relative hepatic *Cd68* gene expression (n=5 mice/group). (d) Relative hepatic *Il6* gene expression (n=5 mice/group). Statistical differences among groups were determined using one-way ANOVA followed by Tukey's post hoc test. Differences were considered significant when *p*-value < 0.05: asterisk indicates *p*-value < 0.05 vs. UN. NAM was dissolved in tap water and provided *ad libitum*. The experiment demonstrates that NAM treatment, particularly at the high dose, reduces hepatic collagen deposition and protects against CDAA-HFD-induced fibrosis. RD, mice fed a standard diet without treatment; UN, mice fed a CDAA-HFD without treatment (untreated); LD, mice fed a CDAA-HFD and receiving a low-dose of NAM (0.25% w/v, dissolved in tap water); HD, mice fed a CDAA-HFD and receiving a

high-dose NAM (1% w/v, dissolved in tap water) (Niño-Narvión J *et al*., 2025, *unpublished data*). The data presented in this study are available on request from the corresponding author.

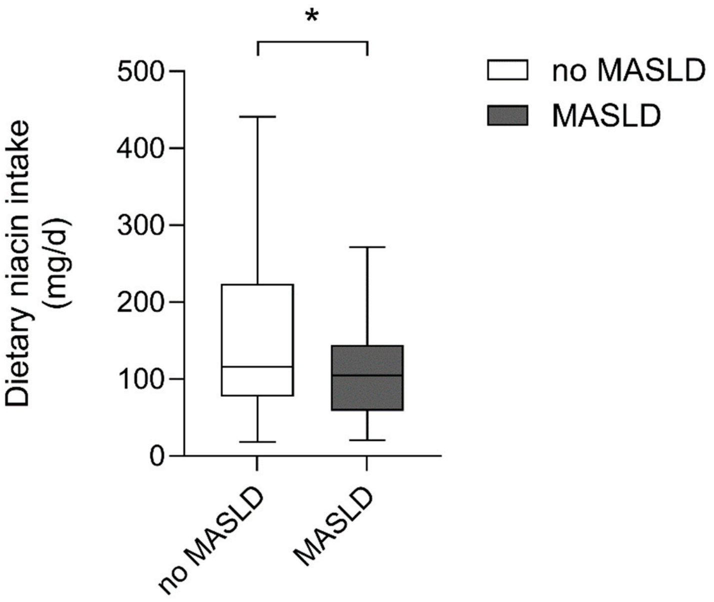

Figure S2. Dietary niacin intake in subjects with morbid obesity. Dietary niacin intake was assessed in morbidly obese individuals (N = 148) undergoing bariatric surgery, including subjects with biopsy-proven metabolic dysfunction-associated steatotic liver disease (MASLD). Dietary intake was evaluated using a validated 136-item Spanish food frequency questionnaire (FFQ). Data are expressed as mean (standard deviation, SD). Patients with MASLD (n = 96) were compared with subjects without MASLD (n = 52). Statistical differences between groups were determined using an unpaired t-test. Differences were considered significant at *p*-value < 0.05; an asterisk indicates *p*-value < 0.05 vs. no MASLD (Martinez-Sanchez, M.A., Ramos-Molina, B. *et al*., 2025, *unpublished data*). The data presented in this study are available on request from the corresponding author.

Supplementary Materials and Methods

Influence of NAM on hepatic fibrosis in mice

Animals and Treatments. All experimental procedures were conducted in accordance with the European Union Council Directives (2010/63/EU) and Spanish Government regulations (RD 53/2013) for the use of animals in research. All efforts were made to minimize animal suffering and reduce the number of animals used. Animal protocols were reviewed and approved by the local Animal Care and Use Committee of our institution (Institut de Recerca de l'Hospital de la Santa Creu i Sant Pau; Procedure Nº 10434), and all methods were conducted according to the approved guidelines. Male C57BL/6JOlaHsd mice (strain code 057; Inotiv, Netherlands) were used. All mice were adults (8 weeks old) at the start of the study and were housed under controlled conditions (20 °C, 66% humidity, 12-h light/dark cycle) with free access to food and water. Mice were exsanguinated directly from the heart at the end of the procedure, and blood was collected into EDTA-containing tubes. Plasma was stored at −70 °C prior to analysis. Livers were removed after euthanasia by cervical dislocation, frozen in liquid nitrogen, and stored at −70 °C or fixed in 10% neutral buffered formalin solution (cat# HT501128, Merck KGaA, Darmstadt, Germany), as appropriate. The same liver lobule was sampled for all analyses in all mice.

Assessment of NAM on Hepatic Fibrosis Induced by a Choline-deficient, L-amino acid-defined high-fat Diet. Hepatic fibrosis was induced by feeding mice a fibrogenic choline-deficient, L-amino acid-defined (CDAA) high-fat diet. Mice were randomly distributed into four groups (n = 5-8 per group) according to the dose of NAM administered in drinking water (0.25% or 0.5%), which was given from the start of the experiment until 6 weeks after fibrosis induction. Untreated mice received no NAM, while a control group (Basal) received normal, non-fibrogenic diet and unsupplemented water. NAM doses were selected based on preliminary experiments that demonstrated their safety in pilot studies. Daily NAM consumption was estimated in individually housed mice from the treated groups. NAM intake was calculated by multiplying the individual daily water consumption by the NAM concentration (%) and expressed as daily uptake per kg of body weight.

Assessment of Hepatic Fibrosis by Sirius Red Staining. Liver tissues were fixed in 4% formalin, embedded in paraffin, and sectioned at 5 μm thickness. Sections were stained with Sirius Red to visualize collagen deposition. Stained sections were scanned using a digital slide scanner, and images were analyzed for fibrosis quantification using Python.

Quantitative Real-Time RT-PCR Analysis. Total RNA was isolated from brain tissue using the TRIzol RNA isolation method (Ambion, cat# 15596018; Life Technologies, Carlsbad, CA, USA) and subsequently purified using the RNeasy Mini Kit Plus (cat# 74134; Qiagen, CA, USA). One microgram (1 μg) of total RNA was reverse-transcribed with Oligo(dT)15 primers using M-MLV Reverse Transcriptase, RNase H Minus, Point Mutant (Promega Corporation, MD, USA) to generate cDNA. Predesigned validated primers (Assays-on-Demand; Life Technologies) and specific TaqMan probes were used. Specific mouse TaqMan probes included Il6 (Mm00446190_m1) and the reference genes Actb (Mm99999903_g1) and Gapdh (Mm99999905_m1). Real-time PCR assays were performed on a C1000 Thermal Cycler coupled to a CFX96 Real-Time System (Bio-Rad Laboratories SA, Life Science Group, Madrid, Spain). All analyses were performed in duplicate, and relative mRNA expression levels were calculated using the ΔΔCt method.

Statistical Analysis. Data are expressed as mean ± standard deviation. The effects of diabetes or NAM treatment on gross and plasma chemical parameters, as well as on relative gene or protein expression levels, were assessed using one-way ANOVA followed by Tukey's post hoc test. Relationships between variables were evaluated using Pearson's correlation. All statistical analyses were performed using GraphPad Prism software (version 5.0, San Diego, CA, USA). A p-value < 0.05 was considered statistically significant.

Dietary niacin intake in individuals with severe obesity undergoing bariatric surgery

Study design and participants. A total of 148 adults with obesity scheduled for bariatric surgery were enrolled at Virgen de la Arrixaca University Hospital (Murcia, Spain) between January 2020 and December 2021. Participants were eligible if they were 18–65 years old, had a BMI of at least 35 kg/m^2, or ≥ 30 kg/m^2 in the presence of obesity-related comorbidities, and a documented history of obesity lasting five years or longer. Individuals were excluded if they presented with any liver disorder unrelated to MASLD, such as viral hepatitis, autoimmune or genetic hepatic diseases, hepatocellular carcinoma, or conditions induced by drugs. Additional exclusion criteria included excessive alcohol intake (>30 g/day for men and >20 g/day for women) or the use of medications known to cause hepatic steatosis (e.g., tamoxifen, amiodarone, valproic acid). All participants provided written informed consent prior to inclusion. Liver biopsies were classified histologically into two groups: (1) non-MASLD, showing normal liver histology; and (2) MASLD, defined by ≥ 5% hepatic steatosis, in the presence of at least one cardiometabolic risk factor. The study followed the principles of the Declaration of Helsinki and was approved by the Ethics and Clinical Research Committees of Virgen de la Arrixaca University Hospital (Ref. 2020-2-4-HCUVA) [152,153].

Dietary niacin intake. The dietary habits of the patients were recorded before surgery by trained dietitians using a semiquantitative Food Frequency Questionnaire (FFQ) previously validated in the Spanish population [154] and comprising 146 food items and 9 consumption frequencies ranging from never or rarely to more than 6 times/day. The recorded intakes were representative of their usual diets. Nutrient intakes were calculated with a computer program designed on the basis of Spanish food composition tables. Dietary niacin intake was also calculated from Spanish food composition tables [155]. Individual intakes were calculated as the mean daily intake (mg/day).

Statistical Analysis. Data were analyzed using GraphPad Prism version 8.0.2 for Windows (GraphPad Software Inc., Boston, MA, USA). The normality of continuous variables was assessed using the Shapiro–Wilk test. Continuous variables are expressed as mean ± standard deviation (SD), whereas categorical variables are presented as frequencies and percentages. Group comparisons (patients with and without MASLD) were performed using the unpaired t-test for normally distributed variables or the Mann–Whitney U test for non-normally distributed variables. The χ^2 test was used to compare categorical variables. Correlations between continuous variables were examined using Spearman's correlation coefficient. Statistical significance was set at p-value < 0.05.
